# Advances and challenges in CAR-T cell therapy for head and neck squamous cell carcinoma

**DOI:** 10.1186/s40364-025-00783-1

**Published:** 2025-05-01

**Authors:** Sahand Saeidpour Masouleh, Kamyar Nasiri, Ava Ostovar Ravari, Mona Saligheh Rad, Kiarash kiani, Ali Sharifi Sultani, Seyedeh Tabasom Nejati, Mohsen Nabi Afjadi

**Affiliations:** 1 Department of Medical Biotechnologies, University of Siena, Siena, Italy; 2 Faculty of Dentistry, Islamic Azad University of Medical Sciences, Tehran, Iran; 3 Faculty of Dentistry, Haybusak University of Medical Sciences, Yerevan, Armenia; 4 Faculty of Dentistry, Iran University of Medical Sciences, Tehran, Iran; 5 School of Dentistry, Hormozgan University of Medical Sciences, Bandar Abbas, Iran; 6 Department of Biochemistry, Faculty of Biological Sciences, Tarbiat Modares University, Tehran, Iran

**Keywords:** Head and neck squamous cell carcinoma, Chimeric antigen receptor T-cell therapy, Immunotherapy, CRISPR

## Abstract

Head and neck squamous cell carcinoma (HNSCC) remains among the most aggressive malignancies with limited treatment options, especially in recurrent and metastatic cases. Despite advances in surgery, radiotherapy, chemotherapy, and immune checkpoint inhibitors, survival rates remain suboptimal due to tumor heterogeneity, immune evasion, and treatment resistance. In recent years, Chimeric Antigen Receptor (CAR) T-cell therapy has revolutionized hematologic cancer treatment by genetically modifying T cells to target tumor-specific antigens like CD19, CD70, BCMA, EGFR, and HER2, leading to high remission rates. Its success is attributed to precise antigen recognition, sustained immune response, and long-term immunological memory, though challenges like cytokine release syndrome and antigen loss remain. Notably, its translation to solid tumors, including HNSCC, faces significant challenges, such as tumor microenvironment (TME)-induced immunosuppression, antigen heterogeneity, and limited CAR T-cell infiltration. To address these barriers, several tumor-associated antigens (TAAs), including EGFR, HER2 (ErbB2), B7-H3, CD44v6, CD70, CD98, and MUC1, have been identified as potential CAR T-cell targets in HNSCC. Moreover, innovative approaches, such as dual-targeted CAR T-cells, armored CARs, and CRISPR-engineered modifications, aim to enhance efficacy and overcome resistance. Notably, combination therapies integrating CAR T-cells with immune checkpoint inhibitors (e.g., PD-1/CTLA-4 blockade) and TGF-β-resistant CAR T designs are being explored to improve therapeutic outcomes. This review aimed to elucidate the current landscape of CAR T-cell therapy in HNSCC, by exploring its mechanisms, targeted antigens, challenges, emerging strategies, and future therapeutic potential.

## Introduction

Head and neck squamous cell carcinoma (HNSCC), a closely related malignancy that affects the oral cavity, pharynx, and larynx, warrants serious attention as it is the sixth most common cancer globally, with approximately 880,000 new cases and 440,000 deaths reported annually [1, 2].

Regarding therapeutic agents, treatment strategies for HNSCC are tailored to tumor stage and molecular characteristics. Despite advances in surgery, radiotherapy, chemotherapy, and immune checkpoint inhibitors, the prognosis remains poor, with a five-year survival rate of only 50–66%. This is primarily due to late diagnosis, high recurrence, and resistance to conventional therapies, driven by factors such as tumor heterogeneity, immune evasion, and treatment resistance [3]. For example, immune checkpoint inhibitors like pembrolizumab and cetuximab show promise but benefit only about 20% of patients due to tumor-induced immune evasion [4]. Of note, HNSCC is marked by aggressive invasion, frequent metastasis, and critical molecular alterations in genes such as TP53, NOTCH1, PIK3CA, EGFR, and CDKN2A [5, 6]. Besides, dysregulated signaling pathways like Wnt/β-catenin, NF-κB, and PI3K/AKT/mTOR contribute to tumor progression and resistance to treatment [7–9], highlighting the urgent need for more effective and targeted therapies.

Given these challenges, chimeric antigen receptor (CAR) T-cell therapy has gained attention as a groundbreaking immunotherapeutic approach for cancer treatment, including HNSCC. This strategy entails the genetic engineering of a patient’s T cells to equip them with a CAR designed to recognize and attack tumor-associated antigens with precision [10, 11]. Unlike traditional immunotherapies, CAR-T cells operate without relying on major histocompatibility complex (MHC) antigen presentation. This unique ability allows them to overcome one of the key immune evasion tactics used by cancer cells, boosting their effectiveness against tumors that inhibit MHC expression [12, 13].

Notably, CAR-T cell therapy has achieved significant success in combating blood cancers, resulting in the FDA approving several CAR-T cell treatments [14]. With these advancements, researchers have turned their focus to exploring CAR-T therapy for solid tumors, including HNSCC. Initial findings indicate that CAR-T cells hold potential in targeting tumor-specific antigens, such as the epidermal growth factor receptor (EGFR), which is commonly overexpressed in HNSCC [10, 15].

Despite its potential, CAR-T therapy for solid tumors faces significant challenges, including the complex tumor microenvironment (TME), tumor heterogeneity, and immune resistance mechanisms. These barriers necessitate continuous advancements in CAR-T cell engineering. The evolution from first- to fifth-generation CAR-T cells has introduced modifications to costimulatory and antigen-binding domains, improving specificity, persistence, and overall therapeutic efficacy [16, 17]. In HNSCC, while clinical trials are ongoing, CAR-T therapy holds promise as a valuable addition to existing treatment strategies, particularly for recurrent and metastatic cases. Additionally, combination approaches integrating CAR-T therapy with other immunotherapies or conventional treatments, such as chemotherapy and radiotherapy, are being explored to enhance treatment effectiveness [18, 19].

Taken together, CAR-T cell therapy marks a significant breakthrough in cancer immunotherapy, offering new hope for patients with HNSCC. However, further research is essential to refine its application in solid tumors, address existing challenges, and enhance clinical outcomes. This review aimed to provide an overview of the current state of CAR-T cell therapy in HNSCC highlighting its mechanisms, targeted antigens, challenges, emerging strategies, and future therapeutic potential.

## Brief pathophysiology of HNSCC

**Fig. 1:**
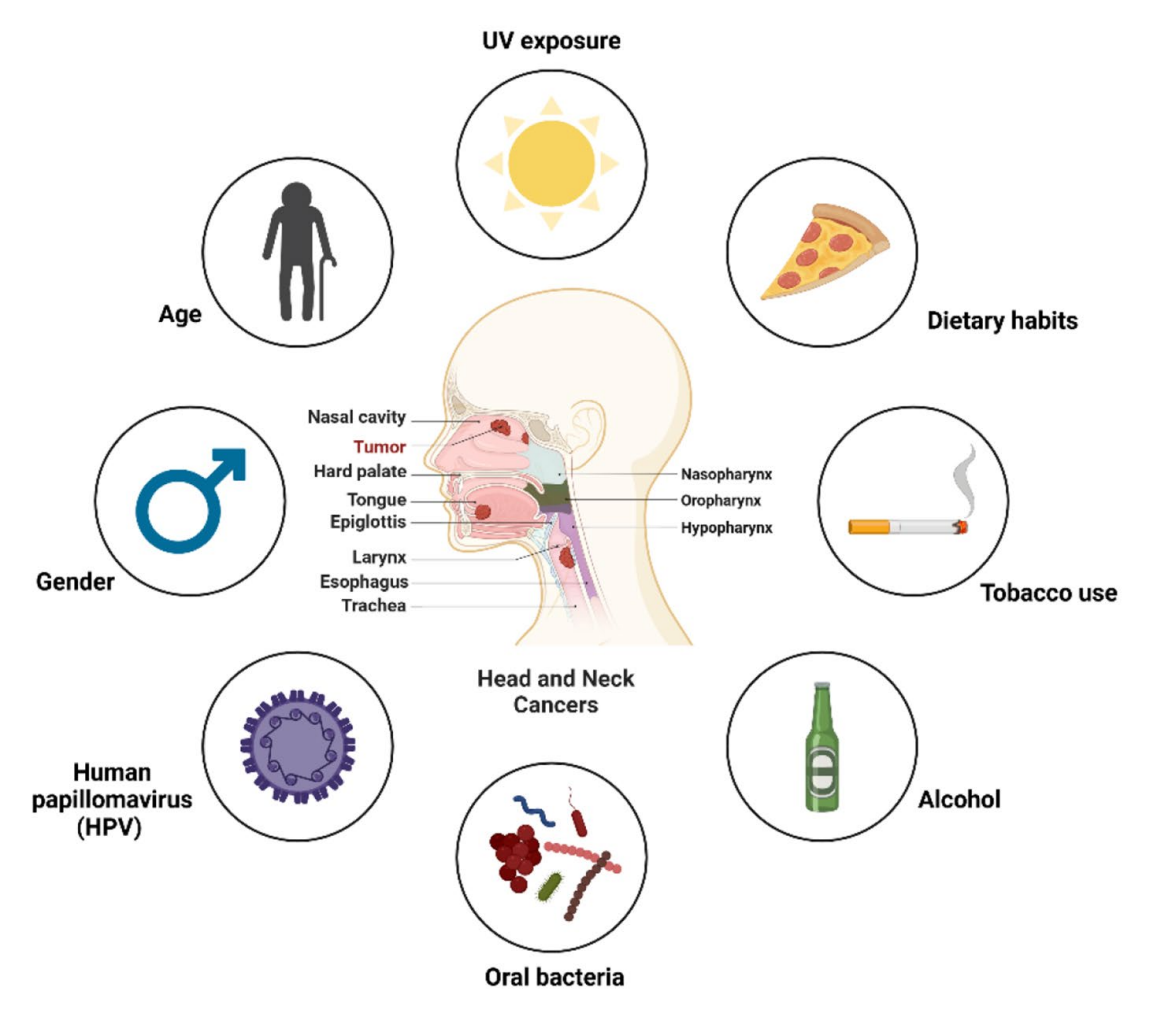
Risk factors related to head and neck cancers. Head and neck cancers are influenced by several risk factors, with tobacco use (smoking and smokeless forms) and excessive alcohol consumption being the most significant. Prolonged exposure to UV radiation increases the risk of lip cancer, while infection with high-risk HPV, particularly HPV-16, is strongly linked to oropharyngeal cancers. Age plays a role, as these cancers are more common in individuals over 50, and men are at a higher risk than women. A poor diet low in fruits and vegetables may contribute to carcinogenesis, while chronic oral infections and certain bacteria, such as *Porphyromonas gingivalis*, may also promote malignancy

HNSCC originates from the mucosal epithelium of the oral cavity, pharynx, and larynx, progressing through a multifaceted interplay of molecular, genetic, and environmental factors. Prolonged exposure to carcinogens, such as tobacco and alcohol, coupled with oncogenic viruses like human papillomavirus (HPV) and Epstein-Barr virus (EBV), triggers genetic instability and disrupts crucial cellular signaling pathways. These elements also contribute to the formation of an immunosuppressive tumor microenvironment (TME), collectively driving the initiation and progression of the disease [1, 20] (Fig. 1).

At the molecular level, HNSCC is characterized by frequent mutations in key tumor suppressor genes and oncogenes [21]. One of the most commonly altered genes is TP53, which encodes the p53 protein responsible for DNA repair and apoptosis. Mutations in TP53 lead to uncontrolled cell proliferation and resistance to programmed cell death [22]. Other critical genetic alterations include mutations in CDKN2A, which disrupts cell cycle regulation, and NOTCH1, which plays a role in cell differentiation [23]. Additionally, oncogenic signaling is often driven by mutations or overexpression of EGFR and PIK3CA [24], activating pathways such as NF-κB, Wnt/β-catenin, and PI3K/AKT/mTOR [7, 25]. These molecular alterations collectively promote tumor growth, survival, and metastasis, contributing to the aggressive nature of HNSCC.

HNSCC is highly invasive and frequently metastasizes to regional lymph nodes [26]. This process is largely driven by the breakdown of the extracellular matrix via matrix metalloproteinases (MMPs) and the induction of epithelial-mesenchymal transition (EMT). In EMT, tumor cells undergo a transformation, shedding epithelial markers like E-cadherin and adopting mesenchymal traits. This shift enhances their ability to move and invade, allowing cancer cells to detach from the primary tumor, penetrate surrounding tissues, and enter the lymphatic system, thereby supporting the spread of the cancer [27].

Along with EMT, angiogenesis is a key factor in the progression of HNSCC. Vascular endothelial growth factor (VEGF) stimulates the formation of abnormal, porous blood vessels that support tumor growth and enable metastasis. These dysfunctional vessels, however, also hinder the infiltration of immune cells and the effective delivery of therapies, contributing to resistance to treatment [28]. These interconnected mechanisms collectively drive tumor progression, highlighting the role of TME in the complexity of HNSCC pathophysiology and the challenges in developing effective treatment strategies.

The TME is composed of a dense stromal matrix, cancer-associated fibroblasts (CAFs), immune cells, and signaling molecules, all contributing to a hostile environment that diminishes the efficacy of anti-tumor immune responses [29]. A key feature of the TME is its capacity to suppress immune responses, enabling the tumor to evade immune surveillance [30]. Immune suppression within the tumor microenvironment is heavily influenced by myeloid-derived suppressor cells (MDSCs), regulatory T cells (Tregs), and tumor-associated macrophages (TAMs) [31]. Tregs secrete cytokines such as TGF-β and IL-10, which inhibit cytotoxic T-cell function and facilitate immune tolerance [32]. MDSCs obstruct T-cell activation and promote angiogenesis, while TAMs, particularly those exhibiting an M2-like phenotype, aid in tumor progression by remodeling the extracellular matrix and suppressing CD8 + T-cell responses [33]. These immune-modulatory mechanisms contribute to the reduced efficacy of immunotherapies, including immune checkpoint inhibitors and adoptive T-cell therapies [34].

T-cell exhaustion is a critical challenge within the TME, resulting from chronic exposure to tumor antigens. This leads to the dysfunction of cytotoxic T cells, marked by the upregulation of inhibitory receptors such as PD-1, CTLA-4, and LAG-3. Exhausted T cells lose their ability to mount sustained immune responses, weakening the body’s natural defense against tumors and diminishing the effectiveness of checkpoint blockade therapies designed to restore T-cell function [34, 35].

In addition to immune suppression, the TME in HNSCC contains CAFs, which also contribute to immune evasion. CAFs facilitate tumor progression and immune escape, creating significant barriers for immunotherapy [29, 36]. Within the TME, FOXP3 + Tregs are characterized by high levels of immune checkpoint receptors, including PD-1 and CTLA-4. Meanwhile, MDSCs contribute to immune suppression by secreting arginase 1 (ARG1) and inducible nitric oxide synthase, which generate nitric oxide. This production of nitric oxide impairs CD8 + T-cell responses, further compromising the immune system’s ability to effectively target and eliminate tumors [37]. ARG1 suppresses T-cell function by reducing CD3ζ-chain biosynthesis, while nitric oxide inhibits activation of key transcription factors like JAK3 and STAT5, further impairing CD8 + T-cell responses [38, 39]. Additionally, macrophages in the TME, particularly TAMs, contribute to immune suppression. TAMs are classified into M1 (pro-inflammatory, anti-tumor) and M2 (anti-inflammatory, pro-tumor) subsets [33, 40], with the M2 phenotype predominating in HNSCC. M2 TAMs promote tumor progression by releasing IL-10 and TGF-β, which further suppress T-cell-mediated anti-tumor immunity [40–43].

On the other hand, tumor growth, metastasis, and resistance to treatment are all influenced by the reciprocal interaction between cancer stem cells (CSCs) TME. By recruiting TAMs, MDSCs, and Tregs, which inhibite anti-tumor immune responses, CSCs actively modify the TME by establishing an immunosuppressive environment. By releasing VEGF and other pro-angiogenic substances, they also encourage angiogenesis and guarantee a supply of nutrients for tumor development. Furthermore, CSCs modify collagen and laminin in the extracellular matrix (ECM), which promotes invasion and metastasis. Through their connections with stromal cells and activation of survival pathways (such as Wnt and Notch), CSCs are further able to cause drug resistance. By activating hypoxia-inducible factors (HIFs) to promote stemness and survival, the TME, on the other hand, provides CSCs with specific niches that enable their self-renewal. Cytokines like PDGF and HGF are secreted by stromal cells, including mesenchymal stem cells (MSCs) and CAFs, which encourage the growth of CSCs. Additionally, the TME protects CSCs from immune monitoring by means of immunosuppressive immune cells and causes metabolic changes in them, such as glycolysis leading to produce lactic acid. As a metabolic byproduct and a signaling molecule that modifies the immunological, stromal, and metabolic landscapes, lactic acid has a complex involvement in the TME and the development of cancer [31, 32].

Notably, although immune checkpoint inhibitors targeting PD-1/PD-L1 and CTLA-4 have shown promise in clinical settings, their effectiveness is often limited by the persistent immune evasion mechanisms present in the TME. On the other hand, the TME also contains potential therapeutic targets that could enhance the success of immunotherapies, particularly CAR-T cell therapies. CAR-T cells can be engineered to specifically target tumor-associated antigens, such as ErbB and MUC1, which are highly expressed in HNSCC. Thus, targeting these antigens may help CAR-T cells overcome immune suppression in the TME and improve their ability to attack and eliminate tumor cells [10, 11, 44].

## Brief introduction to CAR-T cells

CAR-T therapy, developed by Zelig Eshhar and Gideon Gross at the Weizmann Institute of Science in Israel between 1989 and 1993, revolutionized the field of cancer treatment. The technique involves modifying a patient’s own T cells to express CARs, which enable them to specifically recognize and attack cancer cells. This breakthrough approach has significantly boosted the immune system’s capacity to fight cancer. While CAR-T therapy has shown remarkable success in treating blood cancers like leukemia and lymphoma, ongoing research is expanding its potential to target and treat solid tumors as well [45]. The process involves several key steps: (1) the collection of T cells from the patient through leukapheresis or other cell separation methods; (2) activation and transduction of these cells with viral vectors carrying the CAR genes; and (3) expansion of the modified CAR-T cells in vitro before they are infused back into the patient to target and kill cancerous cells [13, 46].

CAR-T cells are engineered with a specialized structure that includes four main components crucial for effective tumor targeting. The CAR consists of several key domains that work together to enhance the T-cell’s ability to recognize and eliminate cancer cells. The antigen-binding domain, derived from antibodies as a single-chain variable fragment (scFv), targets tumor-associated antigens (TAAs) on the surface of cancer cells. The hinge domain provides the necessary flexibility to improve binding efficiency to the tumor. The transmembrane domain anchors the CAR to the T-cell surface, facilitating communication between the extracellular antigen-binding domain and intracellular signaling components. Finally, the intracellular signaling domain, which includes the CD3ζ chain along with costimulatory domains such as CD28 or 4-1BB, triggers the activation of the T cell, prompting a powerful immune response. Together, these components enable CAR-T cells to overcome immune evasion strategies like MHC restriction, allowing for more effective tumor targeting and immune activation [46–49].

**Fig. 2:**
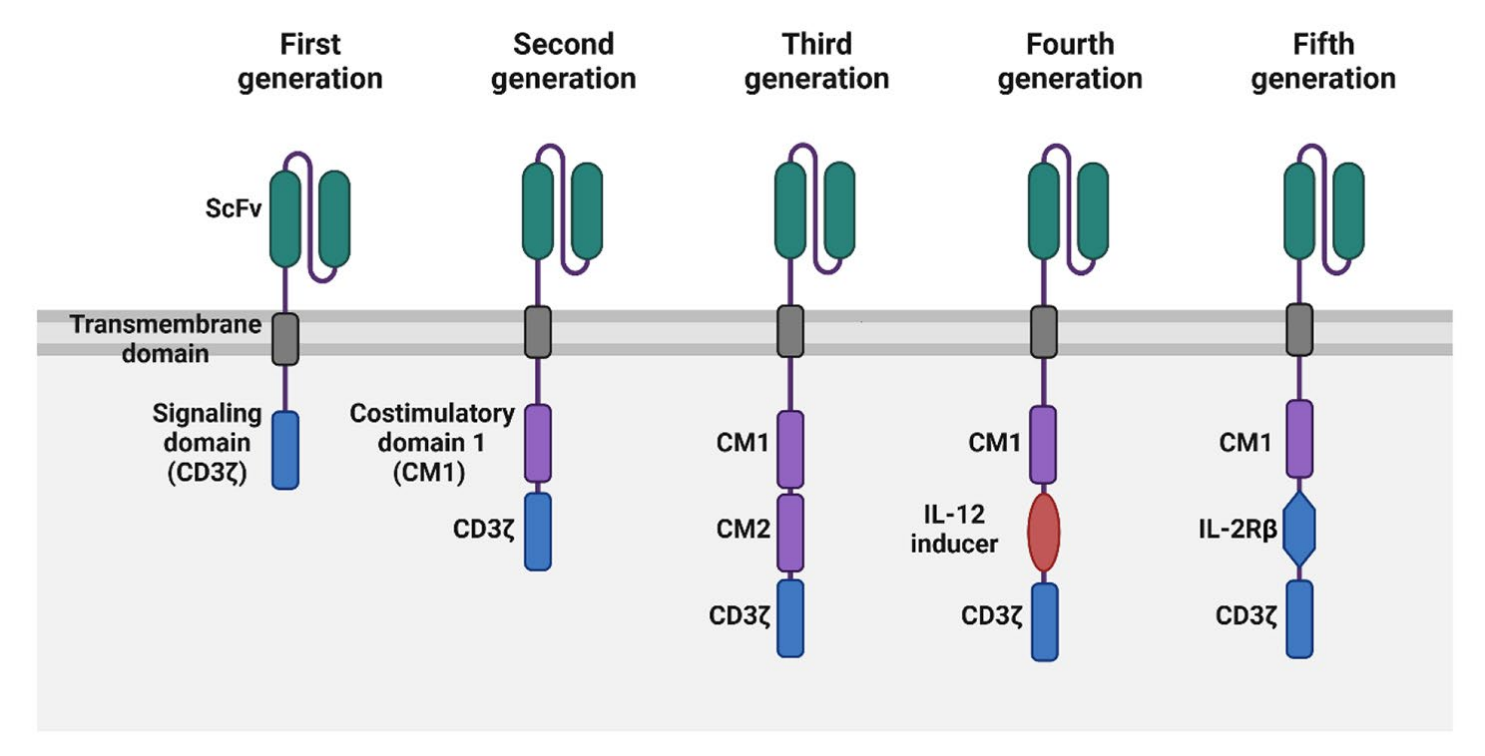
Generations of CAR-T-cell construct designs. CAR T cells have evolved through five generations, enhancing efficacy and persistence. First-generation CARs include only a CD3ζ signaling domain, while second-generation CARs add a co-stimulatory domain (CD28 or 4-1BB) for better activation. Third-generation CARs incorporate multiple co-stimulatory domains, improving proliferation and cytotoxicity. Fourth-generation CARs (TRUCKs) introduce cytokine genes like IL-12 to modify the tumor microenvironment. Fifth-generation CARs integrate cytokine receptor domains (e.g., IL-2Rβ) for enhanced immune signaling. These advancements improve tumor targeting, durability, and adaptability in immunotherapy

In contrast to traditional immunotherapies, CAR-T cells do not depend on MHC molecules for recognizing tumors. Instead, they are genetically modified to specifically target tumor-associated antigens in an MHC-independent fashion. This approach enables CAR-T cells to bypass the immune escape mechanisms typically seen in tumors with low immunogenicity, where conventional immune recognition might fail due to MHC downregulation or loss [12, 50]. The structure of CAR-T cells enables them to selectively bind to tumor cells through the scFv, derived from antibodies that target TAAs. This scFv is linked to the transmembrane and intracellular signaling domains, which play a crucial role in activating the T cells upon antigen recognition, triggering an immune response aimed at eliminating the tumor [50, 51] (Fig. 2).

CAR-T therapy offers a targeted and personalized approach to cancer treatment by using engineered T cells to specifically attack cancer cells, minimizing harm to healthy tissue. This therapy holds the potential for long-term remission and fewer side effects compared to traditional treatments like surgery, radiation, and chemotherapy. Unlike conventional therapies, CAR-T is non-invasive, requires fewer hospitalizations, and can provide durable immune responses, helping to prevent cancer recurrence. This precision approach has transformed cancer treatment, particularly for blood cancers, by harnessing the body’s immune system for a more effective and less damaging fight against cancer [52–54] (Table 1).

**Table 1:** Comparison of CAR-T therapy and conventional cancer treatment

Feature	CAR-T Therapy	Checkpoint Blockade Therapy	Conventional Cancer Treatment (Surgery, Radiation, Chemotherapy)
**Targeting Accuracy**	Selectively attacks cancer cells while minimizing harm to healthy tissue.	systemic targeting of immune checkpoints; less tumor-specific	Impacts both cancerous and normal cells, leading to widespread damage.
**Personalization**	Designed using the patient’s own T cells for a tailored approach.	Low (off-the-shelf monoclonal antibodies)	Uses standardized methods with little individual customization.
**Immune System Effect**	Strengthens the immune response, offering lasting protection against cancer recurrence.	Systemic immune activation; broader effects on immune cells	Requires ongoing monitoring and additional treatments to manage potential relapse.
**Hospitalization**	Fewer hospital visits with a faster recovery process.	Typically, outpatient administration	May necessitate prolonged hospital stays due to treatment complexity and side effects.
**Long-Term Effectiveness**	Can result in sustained remission or potentially a cure.	Durable responses in some cancers (e.g., melanoma) but limited in others	Often demands continuous treatment with limited long-term success.
**Invasiveness**	Administered as a single infusion, avoiding the need for surgery or radiation.	Non-invasive (intravenous monoclonal antibody infusion)	Involves multiple surgical procedures or radiation sessions.
**Side Effects**	Generally milder due to precise targeting.	Moderate to severe (immune-related adverse events: rash, colitis, endocrinopathies)	Can cause severe reactions such as fatigue, nausea, organ damage, and hair loss.
**Cost**	$373,000–$475,000/treatment	$100,000–$150,000/year	$5,000–$50,000/month (chemotherapy); $50,000–$200,000 (radiation/surgery)
**Manufacturing Time**	2–3 weeks (autologous T-cell collection, transduction, expansion)	Lower than CAR-T Therapy	Immediate (off-the-shelf drugs) or days (radiation planning)
**Failur Rate**	30–65% relapse within 1 year (varies by cancer type)	60–80% in tumors with low immune infiltrate or resistance mechanisms	50–90% relapse in advanced cases

CAR-T cells are categorized into different generations based on the development of their intracellular signaling domains. First-generation CAR-T cells included only the CD3ζ chain, which provided the initial signal for T-cell activation. However, these cells showed limited efficacy, as the single signaling domain was not enough to sustain T-cell activation and proliferation. This limitation led to reduced persistence and effectiveness in targeting tumors. To overcome these challenges, second and third-generation CAR-T cells were developed, incorporating additional costimulatory domains to enhance T-cell functionality and improve clinical outcomes [55]. These advancements have significantly improved the persistence, expansion, and therapeutic potential of CAR-T cells [56]. Second-generation CAR-T cells were developed with the addition of costimulatory domains, such as CD28 or 4-1BB, which significantly improved T-cell expansion, persistence, and cytotoxicity [48]. This innovation enhanced the effectiveness of CAR-T therapy, as exemplified by treatments like CTL019 (a second-generation CAR-T), which showed notable success in treating cancers such as relapsed B-cell acute lymphoblastic leukemia (B-ALL). Building on this, third-generation CAR-T cells incorporate multiple costimulatory domains to further enhance T-cell activation [48, 55]. Despite the theoretical advantages of this approach, third-generation CAR-T cells have not demonstrated superior efficacy over their second-generation counterparts in clinical trials [46, 57, 58].

Fourth-generation CAR-T cells, or T-cell redirected for universal cytokine killing (TRUCK), offer a significant advancement in immunotherapy. In addition to targeting and eliminating cancer cells, these modified T cells are designed to secrete pro-inflammatory cytokines, such as IL-12, when activated [48]. This cytokine release not only attracts innate immune cells to the tumor site but also helps break down the tumor’s immunosuppressive environment. Indeed, by amplifying the body’s natural immune response, TRUCK cells hold the potential to improve the effectiveness of CAR-T therapy, particularly in tumors that have developed strategies to avoid immune detection [59]. Early trials have shown promise in improving tumor elimination via the activation of immune cells such as natural killer (NK) and macrophages cells [60, 61].

Fifth-generation CAR-T cells incorporate an IL-2 receptor β (IL-2Rβ) domain, which triggers the STAT3/JAK signaling pathway to enhance their effectiveness. This advancement boosts T-cell survival, proliferation, and tumor-targeting precision, leading to a stronger and more sustained immune response. By optimizing intracellular signaling, these next-generation CAR-T cells are designed to overcome challenges such as immune evasion and tumor-induced suppression, making them a promising option for treating solid tumors where earlier versions have faced limitations [62, 63].

CAR-T cell therapy has revolutionized the treatment of hematological malignancies, leading to the FDA approval of multiple CAR-T products. These therapies have been particularly effective in combating blood cancers such as leukemia and lymphoma, offering a lifeline to patients with relapsed or refractory disease who have exhausted conventional treatment options [64] (Table 2).

**Table 2:** A summary of FDA approval of multiple CAR-T products [64–69]

Generic Name	Trade Name	Target Antigen	Indication	FDA Approval	Mechanism	Patient Population	Clinical Significance
**Axicabtagene ciloleucel**	Yescarta^®^	CD19	Large B-cell lymphoma	Oct 2017	Targets CD19 on B-cells, leading to immune-mediated tumor destruction.	Relapsed/refractory large B-cell lymphoma	First CAR-T for large B-cell lymphoma.
			Follicular lymphoma	Mar 2021	CD19-targeted CAR-T therapy for B-cell malignancies.	Relapsed/refractory follicular lymphoma	Expands CAR-T options for indolent lymphoma.
**Tisagenlecleucel**	Kymriah^®^	CD19	Acute lymphoblastic leukemia	Aug 2017	Targets CD19 on B-cells to treat ALL.	Pediatric/young adult ALL	First CAR-T for pediatric ALL; also approved for B-cell lymphoma.
			Large B-cell lymphoma	May 2018	Treats B-cell malignancies by targeting CD19.	Relapsed/refractory large B-cell lymphoma	Pioneering CAR-T therapy with success in both pediatric and adult cancers.
**Brexucabtagene autoleucel**	Tecartus^®^	CD19	Mantle cell lymphoma	Jul 2020	Targets CD19 on B-cells in mantle cell lymphoma.	Relapsed/refractory mantle cell lymphoma	First CAR-T for mantle cell lymphoma.
**Liscobtagene maraleucel**	Breyanzi^®^	CD19	Large B-cell lymphoma	Feb 2021	CAR-T targeting CD19 for B-cell lymphoma.	Relapsed/refractory large B-cell lymphoma	Expands CAR-T therapy options for relapsed/refractory lymphoma.
**Idecabtagene vicleucel**	Abecma^®^	BCMA	Multiple myeloma	Mar 2021	Targets BCMA on plasma cells in multiple myeloma.	Relapsed/refractory multiple myeloma	First BCMA-targeting CAR-T for multiple myeloma.
**Ciltacabtagene autoleucel**	Carvykti^®^	BCMA	Multiple myeloma	Feb 2022	CAR-T targeting BCMA, enhancing immune response in multiple myeloma.	Relapsed/refractory multiple myeloma	Novel costimulatory domain enhances CAR-T efficacy in multiple myeloma.
**Obecabtagene autoleucel**	Aucatzyl^®^	CD19	Acute lymphoblastic leukemia (ALL)	Nov 2024	Targets CD19 on B-cells, leading to immune-mediated tumor destruction.	Relapsed/refractory B-cell precursor ALL	New CAR-T option for adult ALL; approved based on an open-label, multicenter, single-arm study.

Note: Obecabtagene autoleucel (Aucatzyl) was approved by the FDA on November 8, 2024, as a CD19-targeted CAR-T cell therapy for adults with relapsed or refractory B-cell precursor acute lymphoblastic leukemia (ALL). It is one of the most recent CAR-T therapies to receive FDA approval. Whether Aucatzyl is the seventh approved CAR-T therapy depends on whether any other CAR-T products were approved between Carvykti (February 2022) and Aucatzyl. A full review of FDA approvals during this period would be needed to confirm its exact position in the sequence of approved therapies [65]

Despite the significant success of CAR-T therapy, its clinical application is still hindered by safety concerns. Adverse effects such as cytokine release syndrome (CRS), neurotoxicity, and potential organ damage pose serious risks to patients. These complications arise from excessive immune activation, which, while essential for targeting cancer cells, can also lead to systemic inflammation and unintended tissue damage. Addressing these challenges through improved CAR-T cell designs and optimized treatment protocols remains a key focus in advancing this therapy [70]. As the field evolves, researchers are focused on improving the safety, cost-effectiveness, and versatility of CAR-T therapies. The development of universal CAR-T cells (fifth-generation) represents an exciting advancement that aims to address these challenges and broaden the scope of CAR-T therapy to target a wider variety of tumors [71].

### Mechanisms of killing by CAR-T in HNSCC

CAR-T therapy is an advanced immunotherapy that harnesses genetically modified T cells to precisely target and eliminate cancer. The treatment begins with leukapheresis, a process in which a patient’s T cells are extracted from the bloodstream and sent to a laboratory for genetic reprogramming. Using viral vectors, a synthetic CAR is introduced into these T cells, allowing them to recognize and bind to tumor-associated antigens, such as CD19, which is commonly found on malignant B cells. This modification enhances the T cells’ ability to detect and attack cancer cells independently of MHC presentation, overcoming a key immune evasion strategy used by tumors. After modification and expansion, the CAR-T cells are infused back into the patient, where they proliferate, persist, and actively eliminate cancer cells expressing the target antigen. This approach has demonstrated remarkable success in hematologic malignancies, though researchers continue to refine it for improved efficacy and safety in solid tumors [46, 52, 72, 73]. The CAR structure includes an scFv for antigen recognition, a hinge for flexibility, a transmembrane domain for stability, and intracellular signaling domains (CD3ζ, CD28, or 4-1BB) to enhance activation and persistence. After modification, CAR-T cells are expanded ex vivo and assessed for potency before infusion. Patients receive lymphodepleting chemotherapy (fludarabine and cyclophosphamide) to optimize CAR-T cell expansion and efficacy [13, 73, 74].

Once infused into the patient’s bloodstream, CAR-T cells travel through the body, searching for cancer cells that display the target antigen. When they encounter a tumor cell, the scFv part of the CAR binds directly to the antigen on the cancer cell’s surface. Unlike natural T-cell receptors, CAR-T cells do not need the antigen to be processed and presented by MHC molecules, allowing them to efficiently target tumor cells that might normally escape detection by the immune system. After binding to the antigen, the CD3ζ signaling domain activates, triggering a series of signals that fully activate the T cell. Co-stimulatory signals from molecules like CD28 and 4-1BB further boost the T-cell’s activity, driving its proliferation, cytokine release, and long-lasting persistence, which strengthens the immune response against the tumor [50, 56, 75].

Once activated, CAR-T cells eradicate tumor cells through multiple mechanisms, with the perforin-granzyme pathway playing a crucial role. Upon activation, CAR-T cells secrete perforin, a protein that punctures the tumor cell membrane by forming pores. These openings enable granzyme B, a cytotoxic enzyme, to penetrate the cancer cell, triggering a series of intracellular reactions that culminate in apoptosis, or programmed cell death. This highly efficient process mirrors the body’s natural immune response to malignant cells, enhancing CAR-T therapy’s ability to eliminate tumors effectively [76].

Another important mechanism is Fas-Fas Ligand (FasL) mediated apoptosis, in which CAR-T cells express FasL, which binds to Fas (CD95) receptors on tumor cells, activating the caspase cascade, ultimately leading to programmed cell death [77]. In addition to direct cytotoxic effects, CAR-T cells secrete inflammatory cytokines such as TNF-α, IFN-γ, and IL-2. These cytokines not only enhance the immune response by recruiting additional immune cells, such as NK cells and macrophages, but they also create an inflammatory microenvironment that promotes further tumor destruction [78]. Some CAR-T cells also induce bystander killing, in which cytokine release leads to the destruction of nearby tumor cells that may not directly express the target antigen [78, 79].

## Current applications of CAR-T cell therapy in HNSCC

CAR-T cell therapy has demonstrated significant potential in treating solid tumors, including HNSCC, but faces challenges such as immunosuppressive TME, antigen heterogeneity, poor T-cell infiltration, and physical barriers. Despite these obstacles, recent advances in CAR-T cell engineering have led to innovative strategies aimed at improving efficacy in HNSCC. Current studies focus on novel target antigens, co-stimulatory domains, armored CAR-T cells, and gene editing techniques. However, the transition to clinical trials remains challenging due to the TME and antigen escape. Researchers are developing dual-targeting CAR-T cells, logic-gated CARs, and combination therapies with checkpoint inhibitors to enhance tumor eradication and prevent relapse.

Unlike hematologic malignancies, where CD19-targeted CAR-T therapy has been highly successful, the effectiveness of CAR-T therapy in HNSCC relies on selecting tumor-specific antigens that ensure high tumor selectivity while minimizing off-target effects. The most promising target antigens in HNSCC will be elucidated in the following subheadings.

### ErbB family

In the case of HNSCC, the ErbB family (also referred to as the EGFR family) presents a promising target for CAR-T cell therapy. This family comprises four receptor tyrosine kinases (RTKs)—EGFR (ErbB1), HER2 (ErbB2), HER3 (ErbB3), and HER4 (ErbB4)—which play critical roles in controlling cell proliferation, differentiation, survival, and tumorigenesis. When ligands bind to these receptors, they undergo homo- or heterodimerization, which activates their intrinsic tyrosine kinase activity, setting off a cascade of signaling events that promote cancer progression. Targeting these receptors with CAR-T cells could provide a powerful approach to treating HNSCCs [80, 81]. The activation of ErbB receptors triggers a cascade of downstream signaling pathways that are essential for cellular growth, differentiation, survival, and migration. When these pathways become dysregulated, especially in the case of EGFR, it can drive the pathogenesis and progression of HNSCC. This makes ErbB receptors, particularly EGFR, key targets for targeted therapies, such as CAR-T cell therapy, which could help disrupt these signaling networks and potentially halt tumor growth.

In a preclinical study, CAR-T cells targeting EGFR were evaluated for their potential in treating HNSCC. EGFR-specific CAR-T cells were engineered and tested against FaDu cells, a hypopharyngeal squamous cell carcinoma cell line. The results revealed a significant boost in cytokine release when the CAR-T cells interacted with the tumor cells, signaling robust activation. This cytokine secretion highlighted the CAR-T cells’ ability to recognize and target the cancer cells effectively. Furthermore, the CAR-T cells achieved a 52.66% tumor cell lysis rate, demonstrating their strong anti-tumor effects. These preclinical findings provide evidence that EGFR-targeted CAR-T therapy could be a promising strategy for treating HNSCC, offering targeted tumor destruction while minimizing collateral damage to healthy tissue [82]. However, further studies are needed to assess potential off-target effects, tumor heterogeneity challenges, and the durability of response in clinical settings.

Despite the promising preclinical results, several challenges remain in translating CAR-T therapy from models to clinical settings, particularly due to the heterogeneous nature of solid tumors and the presence of ErbB family members in normal tissues. The expression of these receptors in healthy cells can lead to off-target effects, making it difficult to selectively target tumor cells without damaging surrounding healthy tissue. To overcome these obstacles, researchers have been exploring alternative strategies, such as T4 immunotherapy. This approach aims to refine CAR-T cell specificity and enhance tumor targeting while minimizing adverse effects on normal cells, offering a potential solution to some of the limitations of current CAR-T therapies [83, 84].

T4 immunotherapy is an advanced gene-modified immune cell treatment designed to specifically target ErbB homo- and heterodimers, offering a potential solution to some of the challenges faced by CAR-T therapy in solid tumors. This approach enhances the ability of immune cells to recognize and attack tumor cells that express these specific ErbB receptors, potentially improving treatment outcomes in cancers such as HNSCC. Unlike traditional CAR-T therapies, which may struggle with tumor infiltration and persistence in solid tumors, T4 immunotherapy aims to overcome these hurdles by focusing on precise molecular targets involved in tumor cell proliferation and survival [85]. The T4 immunotherapy approach is a novel strategy that involves modifying patient-derived T cells to express both a chimeric antigen receptor (T1E28z) and a chimeric cytokine receptor (4αβ) via retroviral transduction. The T1E28z CAR is engineered with the T1E ligand, which binds to multiple ErbB dimers, enabling targeted therapy across a range of cancer cells. This CAR incorporates the CD3ζ signaling domain linked to a CD28 hinge, facilitating effective T-cell activation and expansion. In tandem, the 4αβ chimeric receptor combines the ectodomain of IL-4 with the β chain of IL-2/IL-15, promoting T-cell proliferation and enhancing long-term immune responses. thus, by combining these two elements, the strategy seeks to improve CAR-T cell potency, increase their durability, and address the challenges associated with the diversity and complexity of solid tumors [80, 86].

One of the key challenges with T4 immunotherapy is the risk of on-target, off-tumor toxicity, as the ErbB family of receptors are also expressed in normal epithelial tissues. To address this, researchers have explored intra-tumoral delivery of T4-modified T cells, aiming to confine the therapeutic effects within the tumor microenvironment. A Phase I clinical trial, involving 13 patients with advanced HNSCC, has demonstrated that this localized approach is safe. The initial results suggest that intra-tumoral T4 immunotherapy holds promise as a potential treatment for solid tumors. With the trial still ongoing and completion expected by 2025 (NCT01818323), these findings could offer crucial insights into the feasibility and effectiveness of this targeted strategy [87, 88].

In HNSCC, the most frequently mutated genes are TP53 and RAS, both of which play crucial roles in tumor initiation and progression. TP53 mutations disrupt its ability to regulate the cell cycle, leading to unchecked cellular proliferation and reduced apoptotic responses [89]. On the other hand, RAS mutations drive oncogenesis by promoting continuous activation of downstream signaling pathways involved in growth and survival. However, these mutations do not produce unique cell surface antigens, making them unsuitable targets for CAR T-cell therapy. Instead, alternative targets have been explored, with ErbB family members and mucin-1 (MUC1) emerging as promising candidates for immunotherapy [89, 90]. The ErbB family, particularly EGFR, is an attractive target due to its widespread overexpression in multiple solid tumors, including breast, colorectal, and ovarian cancers. In HNSCC, EGFR is overexpressed in more than 90% of cases [80], where its activation triggers dysregulated oncogenic signaling via four major pathways: the Ras/Raf/MEK/ERK-MAPK cascade, which promotes cell proliferation and survival; the PI3K/AKT/mTOR pathway, which enhances cell survival, growth, and metabolism; the PLCγ/PKC axis, which contributes to tumor growth and metastasis; and the JAK/STAT signaling pathway, which mediates immune evasion and promotes chronic inflammation [91]. These pathways collectively drive uncontrolled cell division, evasion of apoptosis, and increased angiogenesis, all of which contribute to tumor aggressiveness.

A promising CAR-T therapy currently in clinical evaluation is the “pan-ErbB-targeted T4 immunotherapy,” which is being tested in a phase I/II clinical trial for HNSCC [80]. This strategy employs a second-generation CAR construct containing a T1E ligand and a 4αβ chimeric cytokine receptor. The T1E ligand is a fusion protein combining the N-terminal of transforming growth factor-alpha (TGF-α) and the C-terminal of EGF, allowing high-affinity binding to EGFR (ErbB1) homodimers and heterodimers, as well as moderate-affinity interactions with ErbB2/3 heterodimers [92]. This broad targeting ensures effective immune-mediated tumor destruction across a range of ErbB-driven tumors. The 4αβ chimeric cytokine receptor is a synthetic receptor that mimics IL-2/IL-15 signaling, enabling enhanced T-cell proliferation and persistence in the hostile tumor microenvironment [80].

However, it should be considered that a key challenge in applying CAR-T therapy to HNSCC is the immunosuppressive nature of the TME. Many HNSCC patients exhibit systemic immune dysfunction, which can hinder the expansion and therapeutic effectiveness of adoptive T-cell therapies. The presence of immunosuppressive factors within the TME significantly reduces the ability of CAR-T cells to effectively target and eliminate tumor cells, limiting the overall success of this promising treatment approach. The 4αβ cytokine receptor in the T4 immunotherapy construct addresses this by delivering a potent IL-2/IL-15-like signal, ensuring the robust expansion and sustained activity of genetically engineered T cells. Additionally, IL-15 signaling plays a crucial role in recruiting and activating NK cells within the tumor microenvironment. NK cells contribute to tumor destruction through multiple mechanisms, including the release of cytolytic granules containing perforin and granzyme, which induce apoptosis in tumor cells, TNF-α signaling, which triggers tumor cell death through pro-inflammatory cytokines, and activation of the TRAIL and FASL death receptor pathways, which initiate programmed cell death in cancerous cells. Indeed, by integrating multi-targeting strategies via the T1E ligand, enhanced persistence via 4αβ signaling, and immune modulation via NK cell recruitment, pan-ErbB-targeted T4 immunotherapy represents a novel and promising approach to overcoming HNSCC resistance to conventional treatments [80, 93, 94].

### CD70

CD70, a TNF ligand, is frequently overexpressed in various solid tumors, including HNSCC. In a study conducted by Park et al., CD70 expression was detected in 19% of HNSCC tumor biopsies. The researchers developed CD70-targeted CAR-T cells using a retroviral human CD70 CAR construct, and these engineered T cells exhibited potent efficacy in targeting and eliminating CD70-positive HNSCC cells. However, the effectiveness of these CAR-T cells is influenced by multiple factors, such as the efficiency of the transduction process, the proportion of CD4 + to CD8 + T cells, and the response to IL-2 stimulation. Variations in these factors across different clinical settings could significantly impact the therapeutic outcome, underscoring the complexity of optimizing CAR-T therapy for HNSCC [95].

These findings were further corroborated by a study in patients with clear-cell renal carcinoma, where infusion of CD70-targeted CAR-T cells resulted in a disease control rate of 76.9%. Remarkably, one patient achieved a long-lasting complete remission, which was still sustained at a two-year follow-up. This outcome highlights the potential of CD70-targeted CAR-T therapy as an effective treatment option for cancers expressing CD70, offering hope for durable responses in certain patient populations [96]. This outcome reinforces the feasibility of using CD70-targeted CAR-T therapy in treating CD70-positive HNSCC, suggesting it may provide a promising new approach for patients whose tumors express this antigen. Given the encouraging results in other cancer types, CD70-targeted CAR-T therapy holds potential for offering durable and effective treatment options for patients with HNSCC, particularly those with tumors that express high levels of CD70. This could pave the way for more personalized and targeted therapeutic strategies in oncology.

The overexpression of CD70 in tumors not only serves as a marker of malignancy but also plays a critical role in establishing an immunosuppressive TME. Elevated CD70 levels are linked to a reduction in CD8 + T cells, which are vital for the immune system’s ability to fight cancer. This depletion of CD8 + T cells facilitates immune evasion, allowing the tumor to persist and grow unchecked. In their study, Park et al. identified nine proteins significantly overexpressed in HNSCC cells, which could serve as potential targets for CAR-T therapy. Among these, anti-CD70 CAR-T cells were found to effectively eliminate HNSCC cells, showing superior efficacy compared to untreated controls. This finding highlights the potential of CD70-targeted therapy as a promising approach for treating HNSCC, offering a new avenue for overcoming the challenges posed by the immunosuppressive TME [95]. These promising results demonstrated that CD70-targeted CAR-T cells can not only enhance tumor elimination but also overcome some of the immunosuppressive challenges typically encountered in solid tumors like HNSCC. Thus, CD70-directed CAR-T therapy represents a potential breakthrough in the treatment of CD70-positive HNSCC, offering hope for more effective immunotherapeutic strategies in the future.

### Mucin-1 (MUC1)

MUC1 represents another compelling target for CAR-T cell therapy, especially in HNSCC, where it is frequently overexpressed. As a member of the mucin family, MUC1 plays a crucial role in maintaining mucosal barrier integrity and promoting cell survival. It achieves this by forming a protective layer on the surface of epithelial cells, which helps shield them from external stressors and contributes to cellular homeostasis. Given its elevated expression in HNSCC, targeting MUC1 with CAR-T cells offers a promising approach to specifically eliminate tumor cells while sparing normal tissues [97, 98]. However, during tumor growth, MUC1 becomes overexpressed due to changes in glycosylation, which induces chronic inflammation and malignant transformation, promoting cancer progression [98, 99].

MUC1 was initially discovered in breast cancer but is also expressed in various epithelial tissues, including the skin, reproductive organs, and immune cells. Studies have shown that CAR-T cells targeting MUC1 can effectively inhibit the growth of triple-negative breast cancer in mouse models, with minimal effects on normal breast epithelial cells. This selective targeting highlights the potential of MUC1-directed CAR-T therapy as a promising treatment option for cancers, such as HNSCC, with reduced off-target toxicity. By focusing on MUC1, CAR-T cells can eliminate tumor cells while sparing healthy tissue, offering a more precise and safer approach to cancer therapy [100].

In HNSCC, MUC1 expression is higher in tumor tissues compared to adjacent normal tissues, and it is associated with poor prognosis, advanced tumor stages, and resistance to radiation therapy. This makes MUC1 an attractive target for CAR-T cell therapy [97, 101]. In a study by Mei et al., the effectiveness of MUC1-targeted CAR-T cells was demonstrated in xenograft models of HNSCC. They went a step further by engineering a fourth-generation CAR-T cell, CAR-MUC1-IL22, which not only targeted MUC1 but also released IL-22. IL-22 is essential for maintaining mucosal barrier integrity by stimulating the production of antimicrobial peptides and mucins in epithelial cells. Additionally, it has pro-inflammatory properties that help activate immune responses. The study showed that IL-22 secretion significantly enhanced CAR-T cell activity, promoting T-cell proliferation, improving long-term survival, and increasing tumor cell killing, thereby offering a promising strategy for improving CAR-T therapy in HNSCC [98].

However, MUC1-targeted CAR-T therapy is not without challenges. Like other CAR-T therapies, MUC1-targeted treatments can cause on-target, off-tumor toxicities because of the low-level expression of MUC1 on normal tissues. This can lead to unintended damage to healthy cells, limiting the therapeutic effectiveness of MUC1-targeted CAR-T cells [102]. To mitigate these potential toxicities and improve the specificity of the therapy, researchers have explored dual-targeted CAR-T cell strategies. This dual-targeting approach leverages the use of a synthetic Notch (synNotch) receptor, which allows CAR-T cells to engage with two distinct tumor-associated antigens (TAAs) simultaneously. Indeed, by requiring two antigens for activation, this strategy ensures more precise targeting of tumor cells while sparing normal tissues that express only one of the targeted antigens [103].

Studies have shown that dual-targeted CAR-T cells, such as those designed to target both MUC1 and ErbB2, can efficiently eliminate tumor cells expressing either MUC1 or ErbB2. This approach improves the specificity of the therapy, allowing CAR-T cells to target a broader range of cancer cells while minimizing off-target effects. By selectively targeting tumor cells that express one or both of these antigens, dual-targeted CAR-T cells reduce the risk of damaging normal cells that may express only one of the antigens, thus enhancing the therapeutic efficacy and safety profile of the treatment [104]. This dual-targeting approach is particularly useful for addressing the challenge of tumor heterogeneity, as seen in cancers like HNSCC, where tumors often escape immune targeting through the loss of specific antigen expression. Thus, by targeting multiple antigens, dual-targeted CAR-T cells may reduce the likelihood of tumor evasion, thus improving the overall efficacy of the therapy [98, 104, 105].

Beyond dual-targeting strategies, suicide genes have been explored as a safety mechanism to control the potential toxicities associated with MUC1- and ErbB-targeted CAR-T therapies. These genetic safeguards allow for the selective elimination of CAR-T cells in the event of severe adverse reactions, such as CRS. A widely used suicide gene in CAR-T therapy is herpes simplex virus-thymidine kinase (HSV-TK), which, when administered alongside ganciclovir, triggers apoptosis in CAR-T cells. This approach provides an essential fail-safe, ensuring that therapy can be halted if toxicity becomes a concern, thereby improving the clinical safety of CAR-T treatments [106]. Another promising suicide gene is the iCasp9 gene, which activates rapid cell death in response to a drug called AP1903. Unlike HSV-TK, iCasp9 offers faster and more controlled activation of cell death, which could improve the management of toxicities like CRS. These suicide genes could play a crucial role in ensuring the safety of MUC1- and ErbB-targeted CAR-T therapies, especially in managing immune-related adverse effects [106–109].

Overall, targeting MUC1 with CAR-T cells offers a promising avenue for HNSCC treatment. The strategy of dual-targeting, such as combining MUC1 with ErbB2, provides a more refined approach, ensuring effective targeting of tumor cells while sparing normal tissues. Additionally, integrating suicide gene technology could further enhance the safety and specificity of CAR-T therapies, mitigating potential risks. Despite these advantages, further clinical research and trials are crucial to fully understand the long-term effectiveness, safety, and overall impact of these advanced therapeutic strategies in the treatment of HNSCC.

### CD98hc

CD98hc (CD98 heavy chain) is a key protein involved in the activation of T cells and the regulation of amino acid transport. Its elevated expression on the surface of radioresistant HNSCC cells has attracted considerable attention as a potential target for cancer immunotherapy. Given its role in promoting tumor cell survival and resistance to radiation, targeting CD98hc with immunotherapeutic strategies, such as CAR-T cells, could help overcome the resistance mechanisms seen in HNSCC. Indeed, by focusing on CD98hc, therapies can selectively target and eliminate resistant tumor cells, enhancing the efficacy of treatment while minimizing damage to surrounding healthy tissues [110]. In a recent study, CD98hc-targeted UniCAR-T cells were developed, showing strong efficacy in eliminating tumor cells in a 3D HNSCC tumor spheroid model. This system demonstrated promising results in both targeting and eliminating HNSCC tumor cells [111].

Moreover, the enhanced infiltration of UniCAR-T cells, driven by the CD98hc-targeted system, plays a crucial role in the success of this therapy. The ability of these engineered T cells to penetrate and effectively target tumor cells is a key factor in achieving therapeutic benefits. Furthermore, the combination of fractionated irradiation with CD98hc-redirected UniCAR-T therapy showed promising synergy. By delivering radiation in smaller, fractionated doses, this approach enhances the effectiveness of UniCAR-T cells, allowing for better tumor infiltration and increased cancer cell elimination. This combined treatment strategy holds significant potential in overcoming the challenge of radioresistance in HNSCC, offering a more potent and durable therapeutic option for patients [111, 112]. Table 3 compare the most promising target antigens for CAR-T therapy in HNSCC.

**Table 3:** Comparison of the most promising target antigens for CAR-T therapy in HNSCC [10, 113–117]

Target	Expression in HNSCC vs. Normal Cells	Advantages	Efficacy	Safety & Side Effects	Off-Target Risks	Selection Criteria
**EGFR**	Overexpressed in 85% of HNSCC (20% very strong). Moderate in normal epithelia.	Well-characterized target with existing therapies. Drives tumor growth and radioresistance.	Preclinical: Enhanced cytokine secretion and tumor lysis. Clinical trials ongoing.	On-target/off-tumor toxicity (skin, gastrointestinal). Cytokine release syndrome (CRS) risk.	Normal epithelial tissues with moderate EGFR.	High tumor expression relative to normal tissues. Established role in tumor progression.
**HER2**	Overexpressed in 19% HNSCC (39% in HPV^+^ oropharyngeal). Low in normal tissues.	Prognostic marker (linked to nodal metastasis and poor survival).	Preclinical: 56% tumor reduction in HER2-targeted CAR-T models.	Limited toxicity in early trials due to low normal tissue expression.	Low risk unless HER2 is aberrantly expressed in non-tumor tissues.	Specificity for tumor cells with minimal normal tissue expression. Prognostic value in HNSCC.
**CD70**	Expressed in 19% of HNSCC tumors (heterogeneous). Rare in normal tissues (activated lymphocytes only).	High specificity in CD70^+^subsets. Synergy with immune checkpoint inhibition.	Efficient killing of CD70^+^ HNSCC cells in vitro. Early trials show 70% response in T-cell lymphoma.	Lymphopenia (due to CD70 on activated T cells).	Activated lymphocytes (temporary depletion manageable).	Limited expression in normal tissues. Potential synergy with other immunotherapies.
**MUC1**	Aberrantly glycosylated MUC1 overexpressed in 80% of HNSCC. Normal MUC1 is membrane-bound; tumor MUC1 is cytoplasmic and truncated.	Tumor-specific epitopes (glycosylation differences). - IL-22-engineered CAR-T enhances efficacy.	IL-22-secreting CAR-T shows stronger cytotoxicity in vitro/in vivo.	CRS and neurotoxicity (common to CAR-T). - IL-22 may exacerbate inflammation.	Normal epithelial MUC1 (low risk due to structural differences).	High expression in HNSCC with tumor-specific epitopes. Potential for IL-22-mediated enhancement.
**CD98hc**	Overexpressed in radioresistant HNSCC. Low in normal tissues (except proliferating cells).	Radiosensitization potential. UniCAR system allows controlled T-cell activity.	Preclinical: Reduced tumor growth in UniCAR models with CD98hc-targeted TMs.	Gastrointestinal toxicity (due to CD98 in gut epithelium).	Proliferating normal cells (e.g., intestinal lining).	Association with radioresistance and potential for combination therapies. Low expression in most normal tissues.

## Challenges and limitations of CAR-T cell therapy in HNSCC treatment

Despite the success of CAR-T cell therapy in treating hematologic cancers, its application in solid tumors like HNSCC remains challenging. One of the biggest obstacles is the TME, which creates a hostile setting for CAR-T cells. The structural makeup of solid tumors, including densely packed cancer cells, stromal barriers, and a rigid extracellular matrix, significantly limits CAR-T cell infiltration. Fibrotic tissue further complicates their movement, reducing the ability of these engineered cells to reach and destroy tumor cells effectively. Addressing these physical and immunosuppressive hurdles is essential for advancing CAR-T therapy in HNSCC and other solid tumors [10, 118–120].

In addition to physical barriers, solid tumors are often associated with harsh physiochemical conditions, including acidic environments, hypoxia, and nutrient deprivation. These conditions are unfavorable for CAR-T cell survival and function, as they can suppress the downregulation of cytokines that are essential for immune cell activation and proliferation. The acidic and hypoxic conditions further hinder the efficacy of CAR-T cells by suppressing their cytotoxic functions and limiting their ability to proliferate and persist within the tumor [10, 118–120].

**Fig. 3:**
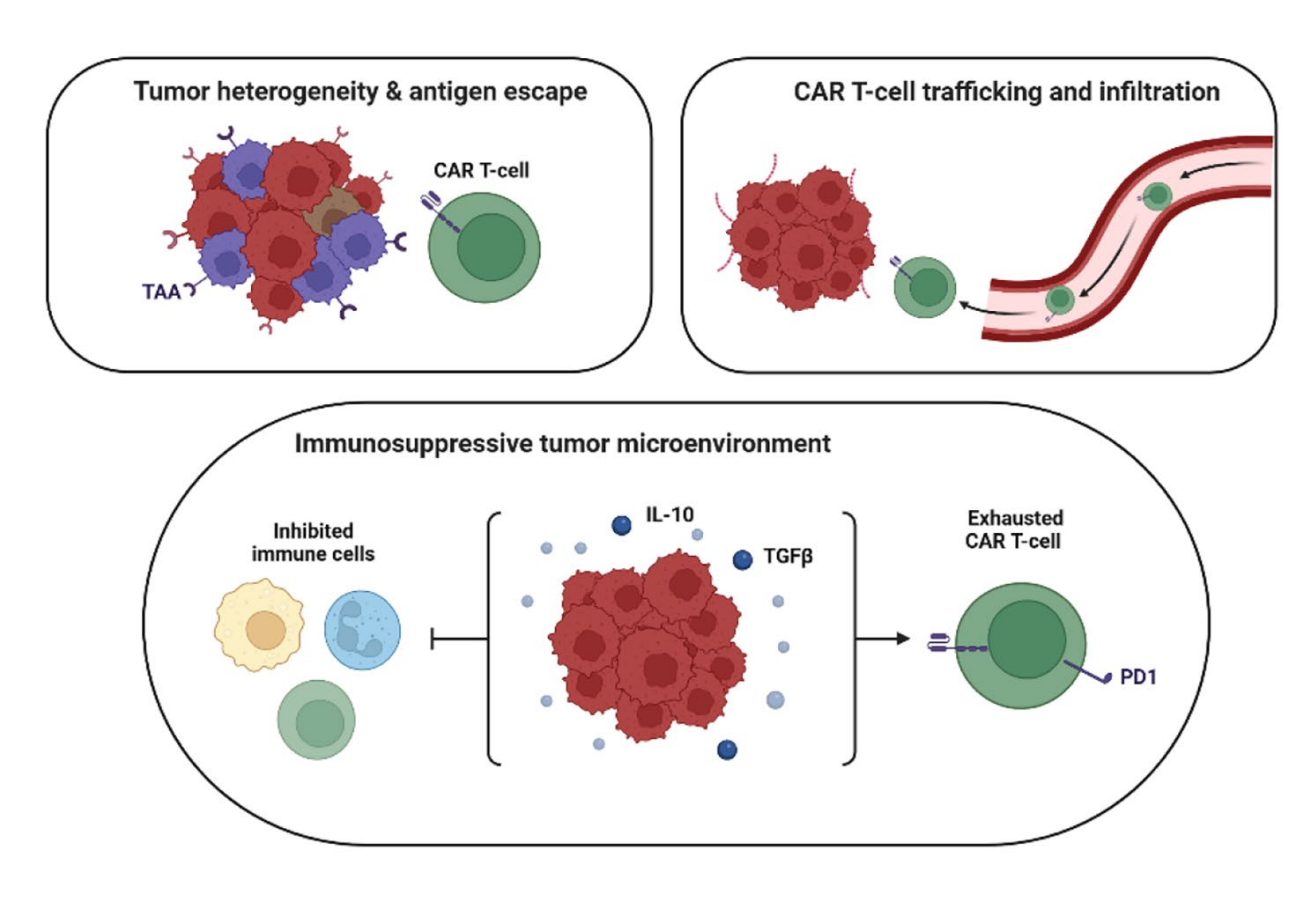
Key challenges in CAR-T cell therapy. CAR-T cell therapy for HNSCCs faces key challenges, including limited infiltration into tumors due to physical barriers in the TME and poor trafficking caused by mismatched chemokine signaling. Tumor heterogeneity further complicates treatment, as diverse TAAs allow cancer cells to evade immune targeting. Additionally, the immunosuppressive TME, dominated by regulatory T cells, myeloid-derived suppressor cells, and inhibitory cytokines like TGF-β and IL-10, diminishes CAR-T cell activity and persistence, significantly limiting therapeutic efficacy

Furthermore, tumors utilize immunosuppressive mechanisms, such as immune checkpoints, to inhibit the immune response. Tumor cells often exploit immune checkpoints like PD-1/PD-L1 or CTLA-4 to suppress CAR-T cell activity, limiting their effectiveness. Tumor antigen loss or heterogeneity further complicates treatment. If a subset of tumor cells does not express the targeted antigens, or if tumor cells undergo antigen modulation, CAR-T cells may fail to recognize and eliminate these cells. The presence of tumor heterogeneity can exacerbate this issue, as CAR-T cells may not effectively target al.l tumor cell populations [10, 118–120] (Fig. 3).

The challenges posed by the TME significantly compromise the infiltration, specificity, and overall effectiveness of CAR-T cells in HNSCC. To overcome these barriers, research focuses on improving CAR-T design and combining it with therapies like immune checkpoint inhibitors, radiation, and targeted treatments. These strategies enhance tumor penetration and sustain CAR-T activity, aiming to make the therapy more effective for solid tumors.

### Barriers to CAR-T cell infiltration in HNSCC

Effective cytotoxic activity of CAR-T cells is contingent upon their successful trafficking to and accumulation at the tumor site. In HNSCC, nonetheless, several barriers hinder this critical process. These include abnormal angiogenesis, which limits blood flow to the tumor, and the dense, fibrous structure of the tumor extracellular matrix that impedes CAR-T cell movement. The limited expression of key chemokines in tumors, along with mismatched chemokine receptors on CAR-T cells, further restricts infiltration. To address this, scientists are enhancing CAR-T cells by equipping them with chemokine receptors that improve tumor targeting. Besides, strategies like modifying the TME with radiation or immune modulators are being explored to break down physical barriers and promote CAR-T cell entry [121–123].

One key approach involves modifying delivery techniques to enhance CAR-T cell migration. While CAR-T cells are typically delivered via intravenous injection, direct intratumoral injection has been explored as a more effective method for solid tumors, including HNSCC. Intratumoral injection of CAR-T cells has been shown to increase their accumulation at the tumor site while minimizing their cytotoxicity to normal tissues [124–127]. In preclinical studies, T1E28z CAR-T cells targeting ErbB + HNSCC demonstrated enhanced therapeutic efficacy when administered via intratumoral injection in mouse models, compared to the conventional intravenous injection. This approach resulted in a more robust antitumor response, likely due to the direct exposure of CAR-T cells to the tumor site, bypassing systemic circulation and potentially overcoming some of the infiltration challenges. Additionally, intratumoral injection was associated with minimal side effects, as no significant CRS or weight loss was observed in the treated mice. These findings suggest that intratumoral delivery may offer a safer and more effective method for administering CAR-T therapy, particularly for solid tumors like HNSCC, by ensuring concentrated treatment at the tumor site while reducing systemic toxicity [127].

Innovative drug delivery systems, including metal-based and biopolymer scaffolds, are emerging as promising strategies to enhance the migration and infiltration of CAR-T cells in solid tumors. These scaffolds, when implanted directly at the tumor site, provide a supportive microenvironment that promotes CAR-T cell survival, persistence, and efficacy. For example, bioengineered polymer matrices that deliver NKG2D-targeted CAR-T cells have shown significant improvements in survival rates in preclinical models, such as those used for ovarian cancer. By creating a more favorable environment for CAR-T cells, these scaffolds help overcome some of the inherent challenges of tumor infiltration and enhance the therapeutic outcomes of CAR-T cell therapy. This approach could potentially be applied to HNSCC as well, helping to address the infiltration barriers and improving the efficacy of CAR-T cells in the treatment of solid tumors [128]. Similarly, biodegradable hydrogel reservoirs, used in a melanoma xenograft model, facilitated the release of CAR-T cells along with anti-PD-L1 antibodies and IL-15 to inhibit tumor growth and prolong survival [129].

In combination with innovative delivery methods, combining CAR-T therapy with other treatments has been proposed to overcome physical barriers in the tumor vasculature. For example, photothermal therapy (PTT) has been used to increase the temperature at the tumor site, which not only kills tumor cells but also reduces the physical barriers in the tumor vasculature, promoting CAR-T cell infiltration. In a melanoma xenograft model, CAR-T therapy combined with PTT resulted in significant tumor growth inhibition [130].

Furthermore, genetically engineered multi-functional CAR-T cells have shown promise in enhancing CAR-T cell infiltration into HNSCC. Indeed, by expressing chemokine receptors like CXCR1 and CXCR2, CAR-T cells can more effectively identify and migrate toward the chemokines expressed by tumor cells. In a glioblastoma xenograft model, genetically engineered CAR-T cells expressing CXCR1 and CXCR2 demonstrated enhanced infiltration and a 100% survival rate in mice, compared to 50% in the control group [131]. Additionally, enzymes like heparanase (HPSE), which degrade components of the tumor extracellular matrix, have been incorporated into CAR-T cells to promote tumor cell migration and enhance the anti-tumor activity of CAR-T cells [132, 133].

These strategies to improve CAR-T cell infiltration, from modified delivery methods to genetic engineering, hold great potential for overcoming the barriers to CAR-T therapy in HNSCC, thus enhancing its clinical efficacy.

### Challenges in the efficacy of CAR-T cells in HNSCC

The efficacy of CAR-T cell therapy in HNSCC is often limited by several factors that can diminish the overall effectiveness of treatment. These barriers include tumor antigen heterogeneity, the tumor’s ability to evade immune responses, the presence of immune checkpoint molecules, and toxicities and cytokine release syndrome related to CAR-T cells therapy.

#### Tumor antigen heterogeneity in HNSCCs

Tumor antigen heterogeneity represents a major challenge in developing effective CAR-T cell therapies for HNSCC, as the antigens expressed by tumor cells can vary significantly across patients and even within the same tumor. These antigens can be classified into four main types: neoantigens, viral antigens, TAAs, and tumor-specific antigens (TSAs). In the case of HPV-related HNSCC, viral antigens, particularly the E6 and E7 oncoproteins, are key targets as they are specific to tumor cells and not typically found in healthy tissue. However, the variability in antigen expression within tumors can lead to immune escape, where certain tumor cells either downregulate or lose expression of the targeted antigen, limiting the effectiveness of CAR-T therapies. To overcome this, strategies like targeting multiple antigens, employing universal CAR-T cell designs, and improving CAR-T cell persistence are being explored to address the issue of antigen heterogeneity and enhance the therapeutic potential of CAR-T in HNSCC [134].

Neoantigens arise from mutations in the tumor genome and are unique to each tumor, which makes them promising targets for therapy but also difficult to generalize across patients. TSAs are unmutated proteins that are either unique or specific to tumor cells, and they are ideal targets for therapies like CAR-T cells [135]. However, neoantigens that are presented by the tumor’s MHC molecules, though suitable for personalized cancer vaccines, can vary greatly between patients, making it difficult to develop a universal CAR-T therapy [136]. TAAs, on the other hand, are antigens that are predominantly expressed on cancer cells but can also be found in smaller quantities on healthy tissues. This characteristic makes them promising targets for CAR-T cell therapy, as the T cells can be engineered to specifically recognize and attack tumor cells. However, the presence of TAAs in normal tissues, albeit at lower levels, increases the risk of unintended collateral damage. As CAR-T cells target these antigens, there is a possibility they may also affect normal cells, leading to off-target toxicity and reducing the overall safety of the treatment [137, 138].

In HNSCC, the problem of antigen heterogeneity is particularly pronounced. The expression of TSAs and neoantigens can differ significantly among patients, making it difficult to create a one-size-fits-all CAR-T therapy. This variability also means that the success of CAR-T therapy may depend heavily on selecting the right target antigen, which is not a straightforward task. Furthermore, targeting TAAs in solid tumors like HNSCC presents an additional challenge, as it can lead to toxicities due to the expression of these antigens in normal tissues. The high antigen heterogeneity within individual tumors also complicates the identification of reliable biomarkers for therapy selection, limiting the development of effective targeted therapies [136–138].

One approach to overcome antigen heterogeneity is to target al.ternative antigens, such as fibroblast activation protein (FAP), which is overexpressed in the tumor stroma of many solid tumors, including HNSCC. FAP-targeted CAR-T cells, when combined with other therapies like radiotherapy, have shown promise in preclinical models by not only enhancing CAR-T cell activity but also remodeling the tumor microenvironment to better support immune response [139]. Clinical trials using FAP-targeted CAR-T cells have demonstrated encouraging results, with minimal toxicity and evidence of in vitro activity against tumor cells. This suggests that FAP-targeted CAR-T cells, especially when combined with other immune therapies, could be an effective strategy for treating HNSCC [140].

Beyond antigen heterogeneity, genetic heterogeneity within tumors poses another challenge. Tumors, including those in HNSCC, often harbor a variety of genetic mutations, leading to the emergence of subclones that may not express the targeted antigens, thus enabling immune escape. This makes it difficult to completely eradicate tumors using traditional CAR-T cell therapy, as tumor cells that do not express the targeted antigen can survive and proliferate. The lack of reliable biomarkers for patient stratification in HNSCC further complicates efforts to develop targeted therapies, as there is no consistent molecular profile to guide treatment decisions.

To address these challenges, researchers are exploring multi-antigen targeting strategies, such as tandem CAR-T cells, which target multiple antigens on the tumor surface. This approach can overcome the problem of antigen escape, where tumor cells may lose the expression of one antigen but still be vulnerable to the attack of CAR-T cells targeting other antigens. In addition, the development of flexible CAR-T cell systems, like the SUPRA CAR system, allows for dynamic reprogramming of CAR-T cells to target different antigens without needing to genetically alter the cells. This adaptability could help manage antigen heterogeneity and enhance treatment efficacy [141, 142]. Another promising avenue is combining CAR-T therapy with oncolytic viruses, which selectively infect tumor cells and express antigens on their surfaces that CAR-T cells can recognize and attack. This combination enhances CAR-T cell targeting and boosts immune responses against the tumor [143, 144].

Genetic engineering of CAR-T cells is a key strategy being explored to enhance their activation, proliferation, and survival in the challenging tumor microenvironment of HNSCC. One promising approach involves modifying CAR-T cells to enhance signaling pathways such as the JAK-STAT pathway, which has been shown to boost T-cell proliferation, improve anti-tumor activity, and increase survival in preclinical models. Additionally, strategies like multi-antigen targeting, where CAR-T cells are engineered to recognize multiple tumor antigens, aim to tackle the issue of tumor antigen heterogeneity. The combination of CAR-T cells with oncolytic viruses, which specifically infect and kill tumor cells, is also under investigation to further enhance tumor targeting and reduce immune evasion. These genetic modifications, when combined with other therapeutic approaches, show significant potential in overcoming the obstacles posed by antigen and genetic heterogeneity, ultimately improving the efficacy and durability of CAR-T cell therapies for HNSCC [143–145]. As these approaches continue to evolve, the potential for CAR-T cell therapy in treating HNSCC becomes increasingly promising.

#### Immunosuppressive TME and poor tumor infiltration in HNSCC

The immunosuppressive TME in HNSCC significantly hampers the effectiveness of CAR-T cell therapies. The TME in HNSCC is characterized by factors such as acidic conditions, hypoxia, low nutrient availability, and the overexpression of immune checkpoints, all of which contribute to immune escape. These conditions inhibit the ability of immune cells, including CAR-T cells, to effectively target and destroy tumor cells. Despite the potential of CAR-T cells to treat HNSCC, their therapeutic efficacy alone has been found to be insufficient, prompting the need for strategies to enhance their activity [146–148].

An innovative approach to enhancing the effectiveness of CAR-T cell therapy in HNSCC involves pre-treating tumors with engineered oncolytic viruses, such as binary oncolytic adenoviruses (CAd). These viruses can be designed to express immune-modulating proteins, including PD-L1 blockade antibodies and cytokines. In HNSCC models, this strategy has shown promise by amplifying the anti-tumor response of HER2-targeted CAR-T cells. The oncolytic virus enhances CAR-T cell function by overcoming tumor-induced immune suppression, improving cell infiltration, and promoting a more robust immune response. This dual approach could offer a powerful tool for increasing CAR-T cell efficacy in challenging solid tumors like HNSCC [149]. In addition to utilizing oncolytic viruses, another promising strategy involves genetically engineering CAR-T cells to secrete specific cytokines that can overcome the immunosuppressive TME. Cytokines such as IL-15, IL-7, IL-12, IL-18, and IL-23 have emerged as critical modulators for enhancing the efficacy of CAR-T cell therapy within the TME. These cytokines are involved in promoting CAR-T cell proliferation, enhancing cytotoxic responses, and improving long-term survival. Thus, by reducing CAR-T cell susceptibility to apoptosis and fostering persistent activity, these cytokines facilitate the sustained targeting and elimination of tumor cells. Incorporating cytokine secretion into CAR-T cell therapy represents a promising strategy to bolster the therapeutic potential of CAR-T cells, particularly for the treatment of HNSCC and other solid tumors [150–153].

The most important strategy for improving CAR-T cell therapy in HNSCC is combination therapy. Incorporating immune-checkpoint inhibitors directly into CAR-T cells is another innovative strategy aimed at overcoming the immunosuppressive TME. Indeed, by engineering CAR-T cells to co-express checkpoint blocking proteins, such as PD-1 decoy receptors or a combination of multiple checkpoint inhibitors like PD-1, Lag-3, and Tim-3, researchers can effectively disrupt the inhibitory signals that suppress CAR-T cell activity. This approach has been demonstrated to significantly enhance the anti-tumor response by preventing immune checkpoint pathways from limiting CAR-T cell function. Moreover, it promotes improved T cell infiltration into the tumor and extends the survival of CAR-T cells, allowing them to persist longer and exert sustained anti-tumor effects. Preclinical models of cancer have shown that this strategy not only amplifies the efficacy of CAR-T therapy but also increases the potential for long-term success in treating solid tumors, such as HNSCC, where immune evasion is a significant hurdle [154]. Oncolytic viruses can enhance CAR-T efficacy by lysing tumor cells and releasing pro-inflammatory cytokines, which recruit immune cells to the tumor site. Cytokine Engineering, Dual-Targeting CAR-T Cells, and CRISPR/Cas9 Gene Editing are other combination strategies to improve CAR-T cell therapy [105].

The TME presents several obstacles to the efficacy of CAR-T cell therapies, including the presence of immunosuppressive cells and factors that restrict immune activity. These include cancer cells, fibroblasts, and various stromal cells that contribute to a hostile environment. A promising strategy to overcome these challenges is the engineering of CAR-T cells to secrete cytokines and chemokines directly within the tumor site. This localized release of immune-modulating factors can boost CAR-T cell infiltration and function within the tumor. Cytokines such as IL-22, IL-18, and IL-12, together with chemokines such as CCL19 and IL-7, play a crucial role in enhancing CAR-T cell activity within solid tumors. These cytokines not only support CAR-T cell proliferation but also amplify their cytotoxic effects. Moreover, by promoting the recruitment of other immune cells, this approach has the potential to significantly enhance the overall efficacy of CAR-T cell therapy in the challenging environment of solid tumors [155]. Furthermore, fourth-generation CAR-T cells, such as CAR-MUC1-IL22 T cells, which secrete IL-22, could enhance the recognition and cytotoxic effectiveness of CAR-T cells in targeting HNSCC. This modification aims to bolster the CAR-T cells’ ability to eliminate tumor cells by providing additional cytokine support, further improving their therapeutic potential in the tumor microenvironment [98].

T-cell exhaustion is another critical challenge in the TME that impairs CAR-T cell efficacy. This exhaustion is often triggered by the activation of the NR4A transcription factor family, which regulates the expression of immunosuppressive molecules like PD-1 and TIM3—markers typically associated with exhausted T cells. Indeed, by knocking out NR4A in CAR-T cells, it is possible to mitigate this exhaustion, resulting in enhanced tumor-killing activity and prolonged cell persistence. This genetic modification could significantly improve the efficacy of CAR-T cell therapies in HNSCC, allowing the CAR-T cells to retain their anti-tumor functions for extended periods. Ultimately, this approach could lead to better therapeutic outcomes by preventing premature T-cell exhaustion in the harsh TME [156].

On the whole, the immunosuppressive TME in HNSCC presents major hurdles for CAR-T cell therapy. However, new strategies are emerging to address these challenges. Modifying CAR-T cells to secrete cytokines, incorporate immune checkpoint inhibitors, or target multiple tumor antigens is enhancing their ability to reach and destroy cancer cells. These breakthroughs offer significant promise for improving the efficacy of CAR-T therapies in HNSCC, providing hope for patients facing this difficult-to-treat cancer.

#### Toxicities related to CAR-T cell therapy in HNSCC

Toxicities linked to CAR-T cell therapy for HNSCC are primarily divided into two categories: “on-target, off-tumor” toxicity and general toxicities. “On-target, off-tumor” toxicity arises when CAR-T cells target antigens that are not only present on cancer cells but also on healthy cells. This can result in unintended damage to normal tissues, potentially causing severe, life-threatening effects, especially when the target antigens are expressed at low levels in non-cancerous cells [157, 158]. To mitigate this issue, the selection of suitable tumor-specific antigens for CAR-T cell therapy and strategies to enhance the selective expression of CAR-T cells at tumor sites are critical areas of ongoing research.

General toxicities are widespread adverse effects arising from the activation of T cells during CAR-T cell therapy, leading to an excessive release of cytokines throughout the body. These toxicities include CRS, macrophage activation syndrome (MAS), hemophagocytic lymphohistiocytosis (HLH), and immune effector cell-associated neurotoxicity syndrome (ICANS). CRS and ICANS are the most severe and unpredictable of these reactions, causing significant complications that require urgent clinical intervention and can severely impact patient safety [159]. CRS is a systemic inflammatory response that typically occurs after the infusion of CAR-T cells. The most common symptom of CRS is fever, which is often accompanied by other signs of inflammation such as fatigue, hypotension, and respiratory distress. CRS is caused by the rapid activation and expansion of CAR-T cells, which subsequently release large amounts of cytokines into the bloodstream. While CRS can range from mild to severe, its management often involves the use of immunosuppressive drugs like tocilizumab, which blocks the effects of IL-6, a key cytokine involved in the response [160].

In addition to fever, other symptoms of CRS can include nausea, fatigue, hypotension, and even cardiac dysfunction. These side effects result from the widespread inflammatory response triggered by CAR-T cell activation. ICANS, which commonly occurs either concurrently with or shortly after CRS, involves neurological complications. ICANS is mainly caused by the disruption of the blood-brain barrier and increased cytokine levels in the cerebrospinal fluid. This cascade of events can result in symptoms such as confusion, encephalopathy, seizures, and, in extreme cases, coma. Treatment typically involves the use of corticosteroids or other immunosuppressive therapies to alleviate the neurotoxic effects and prevent further complications [161]. Notably, ICANS presents with clinical symptoms such as encephalopathy, memory impairment, seizures, speech difficulties, tremors, headaches, language disturbances, and motor weakness. Although CRS is well understood, the exact mechanisms driving ICANS are still not fully elucidated. Researchers continue to investigate how immune cell activation and cytokine release contribute to these neurological symptoms, aiming to improve management strategies for this potentially life-threatening condition [162–164].

CRS in CAR-T cell therapy is characterized by the overproduction of cytokines such as IL-6, TNF-α, IFN-γ, and IL-1, which trigger systemic inflammation and severe adverse events. To address CRS, targeting and inhibiting these cytokines has become a promising therapeutic strategy, particularly for cancers like HNSCC [165]. Approaches include using inhibitors that target the STAT pathway, such as tocilizumab and itacitinib, which help lower cytokine levels and prevent excessive immune activation [160, 166]. Additionally, suicide genes like inducible caspase-9 (iC9) can be employed to counteract toxic effects. Combining CAR-T cell therapy with specific drugs further reduces cytokine release, thereby minimizing the risk of CRS [167–169].

To mitigate the toxicities linked with CAR-T cell therapy, several cutting-edge strategies have been devised. These approaches include multitarget CARs capable of recognizing multiple tumor antigens, affinity-optimized CARs designed for enhanced precision, inhibitory CARs (iCARs) that act as safety switches to control immune responses, and suicide gene incorporation, which allows for the selective elimination of CAR-T cells if needed. A notable advancement by Kosti et al. involves CAR-T cells engineered to detect hypoxic conditions within the tumor microenvironment. These cells selectively express CAR molecules in hypoxic tumor areas, ensuring that they target only the tumor and sparing healthy tissue. This targeted approach minimizes the risk of “on-target, off-tumor” toxicity, offering a safer and more effective treatment strategy for HNSCC and other solid tumors [158].

Another innovative approach is the development of synNotch CAR-T cells by Choe et al., which represent a highly selective form of immunotherapy. These CAR-T cells are engineered with a synthetic Notch receptor that is activated only in the presence of specific tumor antigens, such as EGFRvIII, which is expressed on glioblastoma cells, and myelin oligodendrocyte glycoprotein, found in the central nervous system. This precision targeting allows the CAR-T cells to specifically activate and eliminate tumor cells while minimizing the risk of harming healthy tissues. By ensuring that the immune response is tightly controlled and localized to the tumor, synNotch CAR-T cells hold great promise in enhancing both the safety and efficacy of CAR-T therapy in treating solid tumors like glioblastoma and potentially other cancers with specific tumor-associated antigens [170].

In addition to these strategies, the development of inhibitory CAR-T cells (iCAR-T) presents another approach to mitigating “on-target, off-tumor” toxicity [171]. In iCAR-T cells, the intracellular signaling domain of the traditional CAR-T cells is replaced with inhibitory molecules like PD-1 or CTLA-4. These molecules can dampen the proliferation and cytotoxicity of CAR-T cells when they recognize antigens expressed in normal tissues, thus reducing the risk of unintended tissue damage. This approach holds promise for improving the safety profile of CAR-T cell therapies while maintaining their efficacy in targeting tumor cells [172].

Overall, while CAR-T cell therapy has shown great potential in treating HNSCC, addressing the associated toxicities remains a critical challenge. The development of advanced CAR-T cell designs, including those targeting specific tumor sites and incorporating inhibitory signals, aims to minimize these toxicities and improve the therapeutic outcomes of CAR-T cell therapies in HNSCC and other cancers. Table 4 summarizes the strategies to overcome the challenges in the efficacy of CAR-T cells in HNSCC based on HPV status.

**Table 4:** The strategies to overcome the challenges in the efficacy of CAR-T cells in HNSCC based on HPV status [73, 173, 174]

Barrier	Strategy	HPV^+^ Relevance	HPV^−^Relevance	Example
**Antigen Heterogeneity and toxicity**	Bispecific CAR-Ts (e.g., EGFR + HER2; CD70 + MUC1). TME-activated logic-gated CARs (viral + tumor antigens).	Target HPV E6/E7 antigens and tumor antigens (e.g., CD98hc).	Focus on overexpressed antigens (EGFR, HER2) or TP53 neoantigens.	Bispecific CAR-T trials for HPV + HNSCC (NCT05180216).
	Vaccines (Flt3L + poly(I: C)) + CAR-Ts. Low-dose cyclophosphamide.	Induce epitope spreading via pre-existing T-cell infiltration.	Deplete Tregs to enhance bystander T-cell activity.	Cyclophosphamide used in combination with CAR-Ts (NCT03596086).
**Immune Evasion and Poor Tumor Infiltration**	PD-1/PD-L1 or LAG3/TIGIT inhibitors + CAR-Ts. IL-12/IL-15 armored CAR-Ts.	Counteract T-cell exhaustion (high PD-L1).	Block TGF-β/IDO pathways to reverse immunosuppression.	PD-1 inhibitor + CAR-T trials for HPV + HNSCC (NCT04107142).
	IL-22-secreting CAR-Ts.	Enhance penetration in inflamed TME.	Use IL-15 superagonists (N-803) to sustain CAR-T activity.	IL-22 CAR-Ts in preclinical models (PMID: 36123456).
	Chemokine receptor engineering (CCR2/CXCR2). FAK inhibitors + CAR-Ts.	Leverage chemokines (e.g., CXCL10) from viral infection to recruit CAR-Ts.	Disrupt fibrotic stroma with FAK inhibitors.	FAK inhibitor defactinib + CAR-Ts in trials (NCT03727880).
	Oncolytic viruses (e.g., HSV-1).	Synergize with CAR-Ts via immunogenic cell death.	Lyse tumor cells to release chemokines and recruit CAR-Ts.	HSV-1 + CAR-Ts in preclinical HNSCC models (PMID: 36759523).

## Current status of clinical trials of CAR-T in HNSCCs

CAR-T cell therapy is being used in preclinical and clinical research to treat solid tumors, including HNSCC. Targeted CAR-T cells can reduce tumor size and limit growth of HNSCC cells. The application of CAR-T cell therapy in HNSCC is still in the early stages, with limited clinical trial data available (Table 5).

**Table 5:** Clinical trials of CAR-T therapy

NCT Number / EudraCT	Study Title	Cell Origin	Target Antigen	Cancer Type	Phase	Start Year	Patients	Vector	Sponsors
NCT01818323	T4 Immunotherapy of Head and Neck Cancer	Autologous	ErbB	HNSCC	I/II	2015	30	RV, IT	King’s College London, U.K.
NCT02915445	CAR-T Cells Targeting EpCAM in Advanced Solid Tumors	Autologous	EpCAM	Nasopharyngeal carcinoma, breast cancer	I	2016	30	LV, IV	Sichuan University, China
NCT02980315	EBV-Specific CAR-T Therapy for Malignant Tumors	Autologous	LMP1	EBV + Solid Tumors	I/II	2016	20	LV, IV	Nanjing Medical University, China
NCT03013712	CAR-T Cells for EpCAM + Solid Cancers	Autologous	EpCAM	EpCAM + Solid Cancers	I/II	2017	60	LV, IV	Chengdu Medical College, China
NCT04107142	NKG2DL CAR-Grafted γδ T Cells for Relapsed/Refractory Solid Tumors	Allogeneic	NKG2DL	Relapsed/Refractory Solid Tumors	I/II	2019	10	LV, IV	CytoMed Therapeutics, Malaysia
NCT04249947	P-PSMA-101 CAR-T for mCRPC & Salivary Gland Cancers	Autologous	PSMA	mCRPC, SGC	I/II	2020	60	LV, IV	Poseida Therapeutics, U.S.A.
NCT03740256	Oncolytic Virus + HER2-Specific CAR-T in HER2 + Solid Tumors	Autologous	HER2	HER2 + HNSCC & Solid Tumors	I/II	2020	45	CAdVEC, IT	Baylor College of Medicine, U.S.A.
NCT05239143	P-MUC1C-ALLO1 CAR-T for Advanced/Metastatic Solid Tumors	Allogeneic	MUC1-C	Advanced/Metastatic Solid Tumors	I	2022	100	SB, IV	Poseida Therapeutics, U.S.A.
NCT04847466	PD-L1 CAR-NK + Pembrolizumab in Recurrent/Metastatic HNC	Autologous	PD-L1	Recurrent/Metastatic HNC	II	2021	55	LV, IV	National Cancer Institute (NCI), U.S.A.
EudraCT 2019-004323-20	CLDN6 CAR-T ± CLDN6 RNA-LPX in Relapsed/Refractory Solid Tumors	Autologous	CLDN6	CLDN6 + Relapsed Advanced Solid Tumors	II/III	2020	18	RV, IV	BioNTech Cell & Gene Therapies, Germany
NCT04729543 / NL69911.000.19 and NCT04097301	MAGE-C2 TCR-T for Melanoma & HNSCC	Engineered	MAGE-C2	Melanoma, HNSCC	I/II	2020	-	-	Rotterdam, Netherlands
NCT05117138	CAR-T Therapy for Intermediate & Advanced Tumors	Engineered	AMT-116	HNSCC, NSCLC	Ib/II	2022	-	-	Beijing, China

However, some promising results have been reported. One study involved CAR-T cells targeting ERBb (T1E28z) in HNSCC patients, demonstrating a 69% disease control rate among 12 patients following intratumoral injection [88].

The ongoing clinical trial NCT01818323 focuses on a novel treatment strategy for locally advanced or recurrent HNSCC, known as T4 immunotherapy. This approach involves modifying autologous T cells with retroviral vectors to express two chimeric receptors: T1E28z and 4αβ. The T1E28z receptor specifically targets ErbB dimers, which are frequently overexpressed in HNSCC, while the 4ab receptor promotes T-cell proliferation through activation of key signaling pathways, including STAT3, STAT5, and ERK. Early results from this trial have been encouraging, demonstrating significant antitumor activity in both HNSCC cell lines and mouse models. Notably, the therapy appears to be well-tolerated, with no major toxicity reported, underscoring its potential as a safe and effective treatment for advanced HNSCC [86]. Ongoing research is exploring the combination of T4 immunotherapy with immune checkpoint inhibitors (e.g., anti-PD-1/PD-L1) or chemotherapy to enhance efficacy.

Another trial employed a 3 + 3 dose escalation design, with additional cohorts receiving cyclophosphamide before T4 + T-cell infusion. This cohort also received three doses of nivolumab prior to CAR-T treatment. To address the risk of on-target, off-tumor toxicity from ErbB expression in normal tissues, ErbB-targeted CARs that detect tumor hypoxia offer a promising solution, selectively targeting tumor cells while sparing healthy tissues [87]. Papa et al.’s further evaluation of T4 immunotherapy revealed encouraging results, particularly in advanced HNSCC patients. The study demonstrated a 69% disease control rate following T4 therapy, all without requiring lymphodepletion. The treatment was well-tolerated, with minimal adverse effects (≤ grade 2), and no dose-limiting toxicities were encountered. A remarkable outcome was observed in one patient, who experienced a rapid and complete response after receiving nivolumab in combination with T4. These findings highlight the potential of intra-tumoral T4 administration as both a safe and effective treatment strategy for patients with advanced HNSCC [88].

A Phase I clinical trial, NCT02980315, is examining the safety and effectiveness of EGFR-targeted CAR-T cell therapy in patients with advanced EGFR-positive solid tumors, such as HNSCC. The trial’s objective was to determine whether it would be feasible to use CAR-T cells engineered to target EGFR, a protein that is frequently overexpressed in HNSCC and other solid tumors. The study evaluated the therapy’s maximum tolerated dose (MTD), dose-limiting toxicities (DLTs), and preliminary anti-tumor activity. Initial findings revealed issues like limited CAR-T cell persistence in the tumor microenvironment and on-target/off-tumor toxicity as a result of EGFR expression in normal tissues. Even with these problems, the study taught us a lot about how CAR-T could be used to treat solid tumors and helped us plan the next studies that will combine CAR-T with other methods to make them safer and more effective [175].

The safety and effectiveness of MUC1-targeted CAR-T cell treatment in patients with MUC1-positive advanced refractory solid tumors, such as HNSCC, are being examined by the Phase I clinical study NCT03013712. A glycoprotein called MUC1 is overexpressed in a large number of solid tumors and is linked to both tumor growth and a bad prognosis. The purpose of the trial was to assess the safety, viability, and initial anti-tumor effectiveness of CAR-T cells that were modified to target MUC1. Determining the MTD, evaluating DLTs, and tracking patient growth and persistence of CAR-T cells were among the main goals. The immunosuppressive tumor microenvironment and possible on-target/off-tumor effects are among the difficulties that have been brought to light by the preliminary findings of this and related trials [176].

The safety and effectiveness of CD44v6-targeted CAR-T cell treatment in patients with advanced CD44v6-positive solid tumors, such as HNSCC, are being examined in the Phase I/II clinical research NCT04729543/NL69911.000.19 and NCT04097301. A form of the CD44 protein called CD44v6 is overexpressed in a lot of solid tumors and is linked to treatment resistance, metastasis, and characteristics of cancer stem cells. The purpose of the trial is to assess the safety, viability, and initial anti-tumor effectiveness of CAR-T cells that are designed to specifically target CD44v6. This trial is part of ongoing efforts to expand CAR-T therapy to solid tumors, addressing challenges such as the immunosuppressive tumor microenvironment and target antigen heterogeneity [177].

The safety and effectiveness of PD-L1-targeted CAR-T cell treatment in patients with advanced solid malignancies, such as HNSCC, are being assessed by the Phase I clinical study NCT05117138. An immune checkpoint protein called PD-L1 (Programmed Death-Ligand 1) is frequently overexpressed in solid tumors, which aids in immune evasion. The purpose of this experiment is to evaluate the safety, viability, and initial anti-tumor effectiveness of CAR-T cells that are designed to target PD-L1. In order to increase effectiveness, the study also investigates the possibility of combining PD-L1-targeted CAR-T cells with other immunotherapies or treatments [178].

The safety, tolerability, and effectiveness of autologous iC9-CAR are assessed in Phase Ib/II clinical study NCT06096038. CSPG4 T cells in individuals with HNSCC that is recurrent or refractory. Since HNSCC and other solid tumors overexpress the tumor-associated antigen CSPG4 (chondroitin sulfate proteoglycan 4), it may be a target for CAR-T treatment.

While lymphodepletion is often considered critical in other CAR-T studies for solid tumors, some research suggests that it may not be necessary in all cases. For example, in non-Hodgkin lymphoma patients treated with CD19 CAR-T cells, higher lymphodepletion intensities were associated with improved progression-free survival (PFS) [179]. However, in a study by Wang et al., mesothelin-specific CAR-T cells, generated using CRISPR-Cas9 to delete TCR, were effective without prior lymphodepletion. This highlights the need for further research to understand the role of lymphodepletion in CAR-T therapy for solid tumors like HNSCC [180].

Several clinical trials targeting various antigens in HNSCC are currently recruiting participants. These include studies using intratumoral injections to reduce CAR-T therapy toxicity. Notably, trials investigating CAR-γδT (NCT04107142) and CAR-NK cells (NCT04847466) in HNSCC patients are ongoing. Additionally, a Phase 1/2a trial in Germany is evaluating RNA vaccines combined with CAR-T cells targeting CLDN6 + tumors. Conducted by BioNTech, this study aims to assess the safety and efficacy of CLDN6-targeted CAR-T cells, with or without RNA-lipoplexes (LPX), to potentially enhance the CAR-T cell response by delivering mRNA to antigen-presenting cells (APCs) [181, 182].

In short, while CAR-T therapy for HNSCC is still in early development, initial results are encouraging, particularly with the use of intratumoral injections and combination therapies. However, further research is needed to optimize these treatment strategies and assess the long-term safety and efficacy of CAR-T in solid tumors like HNSCC.

### Strategies to enhance the efficacy of CAR-T cells therapy

Therapeutic strategies to enhance CAR-T cells focus on improving their specificity, efficacy, and persistence in targeting tumor cells while reducing toxicity and overcoming resistance mechanisms.

One of the most promising approaches is combinatorial antigen targeting, which utilizes multiple CARs to increase the density of targetable molecules on the tumor surface. The “OR” strategy involves using multiple CARs that can recognize different antigens, thereby improving the ability of CAR-T cells to locate and eliminate cancer cells. This can be achieved in several ways, including the use of combined CAR-T cell immunotherapy, where two or more CAR-T cell populations are simultaneously or sequentially administered, a strategy known as CARpool [183].

Another promising strategy to enhance CAR-T cell therapy involves the coexpression of two or three distinct CARs within a single T cell, known as dual or triple CAR-T cells. This approach increases the ability of the CAR-T cells to recognize multiple tumor markers, broadening the scope of tumor recognition and improving therapeutic efficacy. Tandem CARs (TanCARs), where two different single-chain variable fragments (scFvs) are linked together, also boost the density of targets on the tumor surface. This increase in target density enhances the potency and antitumor activity of CAR-T cells. A notable example of this strategy is the combination of CAR-CD19-CD28-T cells and CAR-CD19-4-1BB-T cells, which has shown effectiveness in mitigating Trogocytosis—an issue where CAR-T cells lose their antigen recognition capability—using the CARpool method [183].

The issues of antigen heterogeneity and off-target toxicities in cancer immunotherapy might also be greatly addressed by emerging technologies like CRISPR-engineered CAR-T cells and nanobody-based CARs [184, 185]. CRISPR/Cas9 technology is revolutionizing cancer immunotherapy by enabling precise gene editing to overcome critical challenges such as antigen heterogeneity, off-target toxicities, and CAR-T cell exhaustion. Indeed, by knocking out inhibitory checkpoint genes like PD-1 and CD5, CRISPR-engineered CAR-T cells can bypass immune suppression, increasing their proliferation, persistence, and tumor-targeting capabilities. This is particularly important because PD-1 expression typically leads to T cell exhaustion, reducing the effectiveness of the therapy over time [184, 186]. Additionally, CRISPR facilitates the development of “universal” CAR-T cells that can target multiple antigens simultaneously, overcoming tumor heterogeneity—where different tumor cells express varying antigens or tumors evolve and lose targeted antigens. Thus, by targeting multiple antigens, CRISPR-engineered CAR-T cells reduce the likelihood of immune escape, where tumors evade treatment through antigen loss or mutation. Furthermore, CRISPR technology enables the creation of off-the-shelf CAR-T therapies, eliminating the need for patient-specific cell collection and manufacturing, thus reducing treatment costs and improving accessibility. The ability to create these universally applicable CAR-T cells enhances the scalability of cancer immunotherapies, positioning CRISPR/Cas9 as a pivotal tool in advancing more effective, cost-efficient, and durable cancer treatments [88, 187].

Furthermore, compared to conventional scFv-based CARs, nanobody-based CARs provide better antigen specificity and lower immunogenicity because they use smaller antigen-binding domains produced from camelid antibodies. To improve safety and versatility, these nanobody constructions can be designed to target many tumor antigens at once or incorporated into modular systems like SUPRA CARs. When combined, these developments open the door to more accurate and potent CAR-T treatments, reducing side effects and enhancing therapeutic results for solid tumors and hematological cancers [185]. Additionally, nanobody-based CAR-T cells can be combined with immune checkpoint inhibitors or cytokine release, to further enhance the efficacy of treatment while mitigating side effects like cytokine release syndrome (CRS). Their small size and high specificity also reduce the risk of on-target, off-tumor toxicities, as nanobodies are less likely to bind to healthy tissues or activate the immune system inappropriately [188, 189]. Notably, many attempts have been made to use healthy donors’ high-quality T cells to produce allogeneic CAR-T products. Compared to autologous (patient-derived) cells, allogeneic CAR-T cells may be more affordable, more accessible, and produce a higher-quality product [190, 191].

Nevertheless, problems associated with allogeneic cell transplantation, such as host immune system rejection, need to be addressed. A new and exciting development in cellular immunotherapy involves the genetic modification of alternative immune cells, such as γδ T cells, NK cells, Treg cells, Mucosal-associated invariant T cells (MAIT), natural killer T (NKT) cells, natural killer (NK) cells, macrophages, neutrophils, hematopoietic stem/progenitor cells (HSPCs), and induced pluripotent stem cells (iPSCs), to express CARs. These modified cells have shown promising preclinical results and are currently under investigation in clinical trials [192–197].

As of March 2025, there are currently no FDA-approved treatments for oral malignancies, and allogeneic CAR-T cell therapy is still in the early stages of clinical testing. Although obstacles like the tumor microenvironment and antigen heterogeneity still exist, researchers are currently investigating its potential in solid tumors, such as HNSCC. Trials for squamous cell carcinoma and other advanced/metastatic malignancies, such as P-MUC1C-ALLO1, an allogeneic CAR-T treatment that targets tumors that express MUC1C, are being carried out by organizations including UCSF and Moffitt Cancer Center. By providing “off-the-shelf” donor-derived cells, allogeneic techniques seek to overcome the drawbacks of autologous treatments, such as production delays and expenses. Oral malignancies, however, present particular challenges that make CAR design and effectiveness more difficult, including immune suppression and fluctuating antigen expression. Allogeneic CAR-T for oral tumors is still in preclinical or early clinical stages, with continuing research focusing on safety and overcoming solid tumor obstacles, even if pipelines like CRISPR’s CTX112 (now in autoimmune trials) suggest broader possibilities [198–200].

A cutting-edge strategy in CAR-T cell therapy involves the engineering of CAR-T cells to secrete bispecific T-cell engagers (BiTEs), which consist of two single-chain variable fragments (scFvs). One scFv binds to CD3 on T cells, while the other targets a TAA on the tumor cells. This approach enables CAR-T cells to activate and engage not only the engineered T cells but also non-modified, endogenous T cells, effectively boosting the antitumor immune response. Studies have shown that BiTE-secreting CAR-T cells outperform single-target CAR-T cells by targeting multiple antigens and mitigating the risk of tumor escape due to antigen variability. This method recruits additional T cells into the immune response, expanding the range of tumor cells that can be effectively targeted and eliminated [201–205].

Fifth-generation CARs, or universal CARs, are designed to target multiple antigens using adapter elements, allowing a single CAR-T cell population to recognize a broader range of tumor targets. An example of this approach is the SUPRA CAR system. This system comprises a leucine zipper-based CAR (zipCAR) and a scFv that binds to the leucine zipper (zipFv), providing precise control over CAR activation. The system remains inactive until zipFv is present, reducing the risk of unwanted immune responses and offering enhanced flexibility for targeting various tumor antigens [206].

While the “OR” strategy improves antigen recognition, the “AND” strategy enhances safety by ensuring that CAR-T cells only activate when two antigens are simultaneously expressed on the tumor cell. This dual requirement reduces the risk of off-target effects and increases specificity. Early studies demonstrated that CARs could be designed so that CD3z signaling, responsible for cytotoxicity, is triggered by one antigen, while CD28-mediated co-stimulation, essential for proliferation and IL-2 production, is triggered by a second antigen. In dual CAR-T cells co-expressing ErbB2 and MUC1, cytotoxicity depended on ErbB2, while proliferation required both ErbB2 and MUC1. This dual-target system successfully increased specificity, although IL-2 production remained modest compared to traditional CAR-T cells containing both signaling domains [104]. A further refinement of this approach involves synNotch receptors, which allow T cells to recognize a TAA and subsequently express a CAR that activates in response to a second TAA. This system has shown promise in reducing systemic toxicity when applied to solid tumors [207–209]. Additionally, an AND-NOT logic system has been proposed to further minimize adverse effects, in which an iCAR recognizes an antigen found in healthy tissue, preventing CAR-T activation in non-cancerous cells while allowing T cell activation only in the presence of tumor-associated antigens [172].

The success of CAR-T therapy can also be improved through the combination with chemotherapy and radiotherapy. The CAR-T manufacturing process typically takes three to four weeks, which can be critical for patients with aggressive diseases [135, 210]. Bridging chemotherapy is often used to control tumor progression during this period and has been shown to enhance CAR-T efficacy by reducing immune suppressor cells in the tumor microenvironment and upregulating chemokines such as CCL5, CXCL9, and CXCL10 to improve T cell trafficking [211]. However, chemotherapy can also have detrimental effects, as it may reduce the viability of naïve T cells, potentially diminishing the efficacy of CAR-T therapy. Certain chemotherapy agents, such as cisplatin, have been shown to enhance PD-L1 expression in tumor cells via the HSF1-HSP90 axis, which could lead to immune evasion. Therefore, the effectiveness of chemotherapy in combination with CAR-T therapy depends on careful selection of drug dosage and treatment scheduling to maximize benefits while minimizing immunosuppressive effects [211, 212].

Radiation therapy (RT) is another modality that can synergize with CAR-T therapy, particularly in head and neck squamous cell carcinomas, where it is a primary treatment option for nasopharyngeal carcinoma. RT modifies the tumor immune microenvironment by releasing inflammatory mediators that attract immune cells and enhances the exposure of tumor antigens, allowing APCs to capture and present these targets to T cells [213]. The abscopal effect, where local radiation induces systemic antitumor immunity, further supports the rationale for combining RT with CAR-T therapy. Studies have demonstrated that low-dose RT can enhance CAR-T cell infiltration into tumors, increase TCR diversity, and improve CAR-T persistence, making it a promising strategy for overcoming resistance in solid tumors [44, 214].

Recent advancements in mRNA vaccine technology have also opened new avenues for CAR-T therapy enhancement. Originally demonstrated in 1995 [215], mRNA-based immunotherapy can be used to deliver tumor antigens directly to dendritic cells, modify CAR-T cells, or provide a direct source of immunostimulatory molecules. One example is a CAR-T cell-amplifying RNA vaccine targeting the claudin 6 (CLDN6) protein, which has shown promise in treating CLDN6 + lung tumors. This vaccine stimulates CAR-T cell proliferation and persistence while avoiding excessive CRS [216]. Given that CLDN6 expression is significantly elevated in HNSCC, mRNA vaccines may offer a novel means to enhance CAR-T efficacy in these malignancies [217].

## Future directions and conclusion

CAR-T cell therapy, though still in its developmental phase, has shown remarkable potential in treating solid tumors like HNSCC, in addition to its successes in hematological cancers. This therapeutic approach offers hope for patients with refractory and metastatic tumors that do not respond well to conventional treatments. However, challenges persist, such as on-target, off-tumor toxicities, the immunosuppressive tumor microenvironment, cost, manufacturing time, and insufficient T-cell homing to target sites. The translational potential of CAR-T therapy for HNSCC is particularly promising, with several next steps poised to accelerate its clinical application. For instance, clinical trials exploring the combination of CAR-T cells with immune checkpoint inhibitors, such as anti-PD-1 or anti-CTLA-4 antibodies, could enhance therapeutic efficacy by overcoming the immunosuppressive tumor microenvironment. Additionally, integrating CAR-T therapy with oncolytic viruses or metabolic reprogramming techniques may further improve tumor targeting and immune activation.

Critical challenges in solid tumor treatment are tumor heterogeneity and off-target toxicities which may be addressed through personalized CAR-T immunotherapy, use of CRISPR/Cas9 technology, nanobody-based CARs, and allogeneic CAR-T products.

Furthermore, the application of cutting-edge technologies such as MACSima Imaging Cyclic Staining (MICS), introduced by Miltenyi Biotec, represents a breakthrough in personalized CAR-T cell therapy. This immunofluorescent imaging technology enables the identification of hundreds of protein targets from a single specimen, facilitating the discovery of patient-specific antigens for CAR-T cell design. The integration of such advanced techniques into CAR-T therapy could pave the way for fully personalized cancer immunotherapies, enhancing precision and therapeutic success [218].

As CAR-T technology advances, the landscape of cancer immunotherapy is poised to shift with the development of more advanced strategies. These may include universal CARs, which can target multiple antigens across a variety of cancers, and multiplexed gene editing to simultaneously manipulate several genes for improved efficacy. Additionally, the combination of CAR-T cells with immune checkpoint inhibitors, oncolytic viruses, or metabolic reprogramming techniques could offer synergistic effects, enhancing therapeutic outcomes. With ongoing research and clinical validation, CAR-T therapy holds the potential to transform the treatment of solid tumors, bringing renewed hope to patients facing cancers that were once deemed untreatable.

In conclusion, future clinical trials should focus on optimizing CAR-T cell homing, persistence, and safety in HNSCC patients, while also evaluating the long-term efficacy of combination therapies. By addressing these challenges and leveraging the full potential of CAR-T technology, the field is poised to transform the treatment landscape for HNSCC and other solid tumors, offering renewed hope to patients with refractory and metastatic disease.

## Data Availability

No datasets were generated or analysed during the current study.

## Abbreviations

HNSCC: head and neck squamous cell carcinoma
CAR: chimeric antigen receptor
MHC: major histocompatibility complex
TME: tumor microenvironment
HPV: human papillomavirus
EBV: Epstein-Barr virus
MMPs: matrix metalloproteinases
EMT: epithelial-mesenchymal transition
CAFs: cancer-associated fibroblasts
Tregs: Regulatory T cells
MDSC: myeloid-derived suppressor cells
TAMs: tumor-associated macrophages
scFv: single-chain variable fragment
TAA: tumor-associated antigens
TRUCK: T-cell redirected for universal cytokine killing
NK: natural killer
CRS: cytokine release syndrome
MUC1: mucin-1
TGF-α: transforming growth factor-alpha
TSAs: tumor-specific antigens
FAP: fibroblast activation protein
